# Effectiveness of Mobile Health Interventions in Pediatric Cancer: Systematic Review and Meta-Analysis of Randomized Controlled Trials

**DOI:** 10.2196/86836

**Published:** 2026-04-22

**Authors:** Huilu Yao, Yiting Wen, Hongxiu Wang, Ying Xiao, Meiling Deng, Wei Yang, Yuqin Li, Xiaorong Mao

**Affiliations:** 1 College of Nursing, Chengdu University of Traditional Chinese Medicine, Chengdu, China; 2 Department of Nursing, Sichuan Provincial People’s Hospital, School of Medicine, University of Electronic Science and Technology of China, No. 32, West Section 2, 1st Ring Road, Qingyang District, Chengdu, Sichuan Province, 610072, China, 86 13551093555, 86-028-87795585; 3 School of Medicine, University of Electronic Science and Technology of China, Chengdu, China; 4 College of Nursing, North Sichuan Medical College, Nanchong, China; 5 Department of Neurosurgery, Sichuan Provincial People’s Hospital, School of Medicine, University of Electronic Science and Technology of China, Chengdu, China; 6 Department of Thoracic Surgery, Sichuan Provincial People’s Hospital, School of Medicine, University of Electronic Science and Technology of China, Chengdu, China; 7 Department of Cancer Center, Sichuan Provincial People’s Hospital, School of Medicine, University of Electronic Science and Technology of China, Chengdu, China

**Keywords:** pediatric cancer, mobile health, adherence, quality of life, meta-analysis

## Abstract

**Background:**

Cancer poses a significant threat to children’s health, and mobile health (mHealth) is emerging as a key tool for remote disease management, health education, and follow-up. However, evidence of its effectiveness remains limited.

**Objective:**

This study aimed to summarize the effects of mHealth interventions for pediatric cancer compared with usual care, providing evidence-based support for optimizing intervention models and improving patient outcomes.

**Methods:**

A systematic search of 14 databases identified randomized controlled trials (RCTs) on mHealth apps for pediatric patients with cancer from inception to August 1, 2025. Two reviewers independently screened studies, extracted data, assessed bias risk, and graded evidence quality. The meta-analysis was conducted using RevMan 5.4 and Stata 15.

**Results:**

A total of 24 RCTs involving 2645 patients were included. This review found that mHealth interventions significantly reduced infection rates (odds ratio [OR] 0.25, 95% CI 0.10-0.60; *P*=.002) and the overall incidence of peripherally inserted central catheter (PICC) complications (OR 0.16, 95% CI 0.10-0.24; *P*<.001), while improving quality of life (standardized mean difference [SMD] 1.34, 95% CI 0.13-2.55; *P*=.03), self-management ability (SMD 6.39, 95% CI 1.26-11.53; *P*=.01), and treatment adherence (OR 2.83, 95% CI 1.41-5.66; *P*=.003). However, mHealth interventions had no significant effect on PICC catheter displacement (OR 0.44, 95% CI 0.15-1.29; *P*=.13) or health knowledge (SMD 4.44, 95% CI −2.40 to 11.29; *P*=.20). Further high-quality studies are needed to verify their impact in these areas. The intervention components covered 9 behavior change techniques: goals and planning, feedback and monitoring, social support, shaping knowledge, repetition and substitution, reward and threat, comparison of outcomes, natural consequences, and regulation.

**Conclusions:**

This systematic review and meta-analysis synthesized evidence from RCTs. The findings support the use of mHealth to reduce infections and PICC-related complications among pediatric patients with cancer while improving quality of life, self-management capabilities, and treatment adherence. These results underscore the importance of incorporating mHealth strategies into pediatric cancer care and guide the development and enhancement of future mHealth interventions.

## Introduction

Cancer remains a major threat to the health and survival of children [1]. While advances in medical technology have improved survival rates [2], children living with cancer continue to face considerable challenges. First, the disease itself compromises immune function and causes neutropenia, increasing susceptibility to bacterial, fungal, and viral infections [3], which is one of the leading causes of mortality in this population [4]. Second, following diagnosis, pediatric patients with cancer typically undergo intensive treatments. Chemotherapy, used in over 95% of pediatric cancer cases [5], is associated with adverse effects such as pain, vomiting, and fatigue, which substantially diminish quality of life (QoL) [6]. Additionally, pediatric patients typically require the placement and maintenance of a peripherally inserted central catheter (PICC) during chemotherapy, yet PICC-related complications—including bloodstream infections, venous thrombosis, and mechanical issues—remain frequent. These complications can delay antitumor therapy, prolong hospitalization, and increase mortality risk [7]. The complexity of disease management, coupled with limited access to routine care information [8] and frequent care transitions between hospital and home [9], places a heavy burden on pediatric patients and their families. Therefore, timely and effective health education and self-management support are crucial.

In this context, mobile health (mHealth) has emerged as a key tool in health care interventions due to its convenience, efficiency, accessibility, and low cost. mHealth has been shown to overcome barriers related to limited human resources and distance [10]. The World Health Organization defines mHealth as the use of mobile information technology devices, such as mobile phones, tablets, wearable devices, and wireless pedometers, in medical and public health practices [11]. It enables remote services, including symptom monitoring, health education, vital sign tracking, alerts, and medication guidance [12,13].

Previous reviews have examined mHealth apps in pediatric cancer care. Ramsey et al [14] found that mHealth can reduce pain and improve treatment adherence, though findings regarding health-related QoL were inconsistent. González-Díaz et al [15] observed that mobile apps effectively reduced the incidence and severity of symptoms, such as pain and nausea, with high usability and acceptance among patients and caregivers. Similarly, Upreti et al [16] noted that pain monitoring apps reduced pain intensity and decreased moderate-to-severe pain episodes, demonstrating good usability and satisfaction. Zhu et al [17] concluded that mHealth enables multidimensional pain assessment (intensity, frequency, location, and associated symptoms) with a favorable user experience. Delemere et al [18] found that incorporating high-frequency behavior change techniques (BCTs), such as feedback, monitoring, and social support, in pediatric cancer-related mHealth apps enhances treatment adherence and caregiving coping abilities. Mehdizadeh et al [19] further observed that smartphone apps improve symptom reporting adherence, health care communication, and medication compliance, with good user acceptance.

However, existing reviews have several limitations. First, most analyses included primarily pilot or small-sample exploratory studies, with no comprehensive synthesis of randomized controlled trials (RCTs) on mHealth apps for pediatric patients with cancer. Second, outcomes have often focused on mHealth device usability and pain management, lacking thorough evidence on clinical effectiveness in areas such as infection prevention and PICC care [18]. Third, some reviews combined children and adolescents in their analyses, resulting in high sample heterogeneity. These reviews also failed to distinguish between mHealth users (children or caregivers) and treatment stages (treatment or recovery phases) [20], making it difficult to rule out confounding factors influencing outcomes [15]. This limits the specificity and applicability of the evidence. Fourth, prior reviews have largely been restricted to English-language databases and evidence from high-income countries, offering limited insight into mHealth effectiveness in low-income and middle-income regions [15,19]. Furthermore, many existing mHealth platforms lack clinical and evidence-based input in their design, underscoring the need for high-quality evidence to guide development [21].

To address these limitations, this study conducted a systematic search of Chinese and English databases, aiming to synthesize the effects of mHealth interventions in children aged 0‐18 years undergoing cancer treatment by using meta-analyses based on RCTs. The primary outcomes are infection incidence, QoL, and PICC-related complications. Secondary outcomes include health knowledge, self-management ability, and treatment adherence. The BCT taxonomy was used to categorize intervention components across studies, thereby providing more practice-oriented evidence for mHealth apps in pediatric cancer care.

## Methods

### Ethical Considerations

This study did not involve the collection of primary data from human participants. All data were derived from previously published sources and are appropriately cited. Therefore, ethical approval was not required.

### Study Registration

This review was registered with PROSPERO (registration number: CRD420251108938). During the review process, this study strictly adhered to the registered protocol. In the initial search strategy, broad telehealth-related terms were applied to comprehensively assess the multidimensional effects of telehealth interventions on pediatric patients with cancer. However, during the systematic search and screening process, it was observed that most (over 90%) of the studies meeting the original inclusion criteria used mHealth-based interventions—such as mobile apps, web-based platforms, or smart devices—rather than traditional telehealth approaches (eg, telephone follow-ups or video consultations) [22,23]. Meanwhile, a sufficient number of RCTs were identified. To enhance internal homogeneity and ensure that the review provides precise, high-quality evidence with clear practical implications, the scope of this review was refined to focus exclusively on RCTs of mHealth interventions. The registration record has been updated accordingly, and the systematic review and meta-analysis were conducted in strict accordance with the most recent registration record [24].

### Search Strategies

The systematic review and meta-analysis were conducted in accordance with the PRISMA (Preferred Reporting Items for Systematic Reviews and Meta-Analyses) 2020 statement [25] (Checklist 1). A comprehensive search was conducted across PubMed, Web of Science, Cochrane Library, Embase, CINAHL, Scopus, ScienceDirect, ProQuest, PsycINFO, OVID, Chinese National Knowledge Infrastructure, WanFang, Weip Database, and China Biology Medicine database, with each searched from inception to August 1, 2025. The search terms combined both Medical Subject Headings (MeSH) terms and free-text keywords: (cancer or oncology or tumor or neoplasm or leukemia) and (child or pediatric) and (mHealth or internet-based intervention or software application). The detailed search strategy is provided in Multimedia Appendix 1.

### Study Selection and Data Extraction

Two researchers (HY and YX) first removed duplicates using EndNote X21 (Clarivate Analytics) and then screened the studies in 2 steps: first by title and abstract, and then by full text. Any discrepancies that arose during the screening process were resolved through discussion between the 2 researchers, or, if necessary, by consulting a third researcher (XM). The literature screening followed the participants, intervention, comparison, outcomes, and study design [26]. Inclusion criteria were as follows:

1. Participants: children aged 0‐18 years diagnosed with cancer
2. Intervention: mobile-based or wireless-based tools such as apps, websites, wearables, or social media
3. Comparison: usual care (paper-based education or face-to-face monitoring)
4. Outcomes: infection incidence, QoL, PICC-related complications, health knowledge, self-management ability, and treatment adherence
5. Study design: RCTs

Exclusion criteria included (1) reviews, abstracts, theses, systematic reviews, meta-analyses, and case reports; (2) unavailable full texts; (3) interventions targeting only parents; (4) publications not in Chinese or English; and (5) interventions limited to calls or videos.

Two researchers (HW and YW) independently extracted the following data using a standardized form, including the first author, publication year, country, sample size, age, sex, cancer type, intervention duration and setting, mHealth platform, BCT clusters, intervention control groups, and outcome measurement tools.

### Quality Assessment

Two researchers (HY and YW) independently assessed the methodological quality of the included studies using the revised Cochrane Risk of Bias 2 tool, evaluating selection, performance, attrition, detection, and reporting biases, each rated as low, some concerns, or high [27]. The Grading of Recommendations Assessment, Development, and Evaluation (GRADE) framework was applied to appraise the quality of evidence using GRADEpro. Results were categorized into 4 levels (high, moderate, low, and very low) according to predefined criteria. The assessment incorporated study design, risk of bias, inconsistency in population, heterogeneity of findings, statistical precision of effect estimates, and publication bias [28]. Discrepancies were resolved through discussion or, if needed, consultation with a third researcher (XM).

### Statistical Analysis

This study adhered to the Cochrane Handbook [29] for data synthesis and analysis, using RevMan 5.4 (Nordic Cochrane Center) and Stata 15.0 (StataCorp) for statistical procedures. Effect measures included standardized mean differences (SMD, 95% CI) and odds ratios (OR, 95% CI). Heterogeneity was assessed using *I²* and *P* values; when *I²* >50% and *P*<.1 (indicating substantial heterogeneity), a random-effects model was used; otherwise, a fixed-effects model was used. Sensitivity analyses were conducted using a stepwise exclusion, while subgroup analyses and meta-regression were used to explore heterogeneity. Publication bias was assessed with funnel plots and Egger’s test. A *P*<.05 was considered statistically significant.

## Results

### Search Results

**Figure 1.**
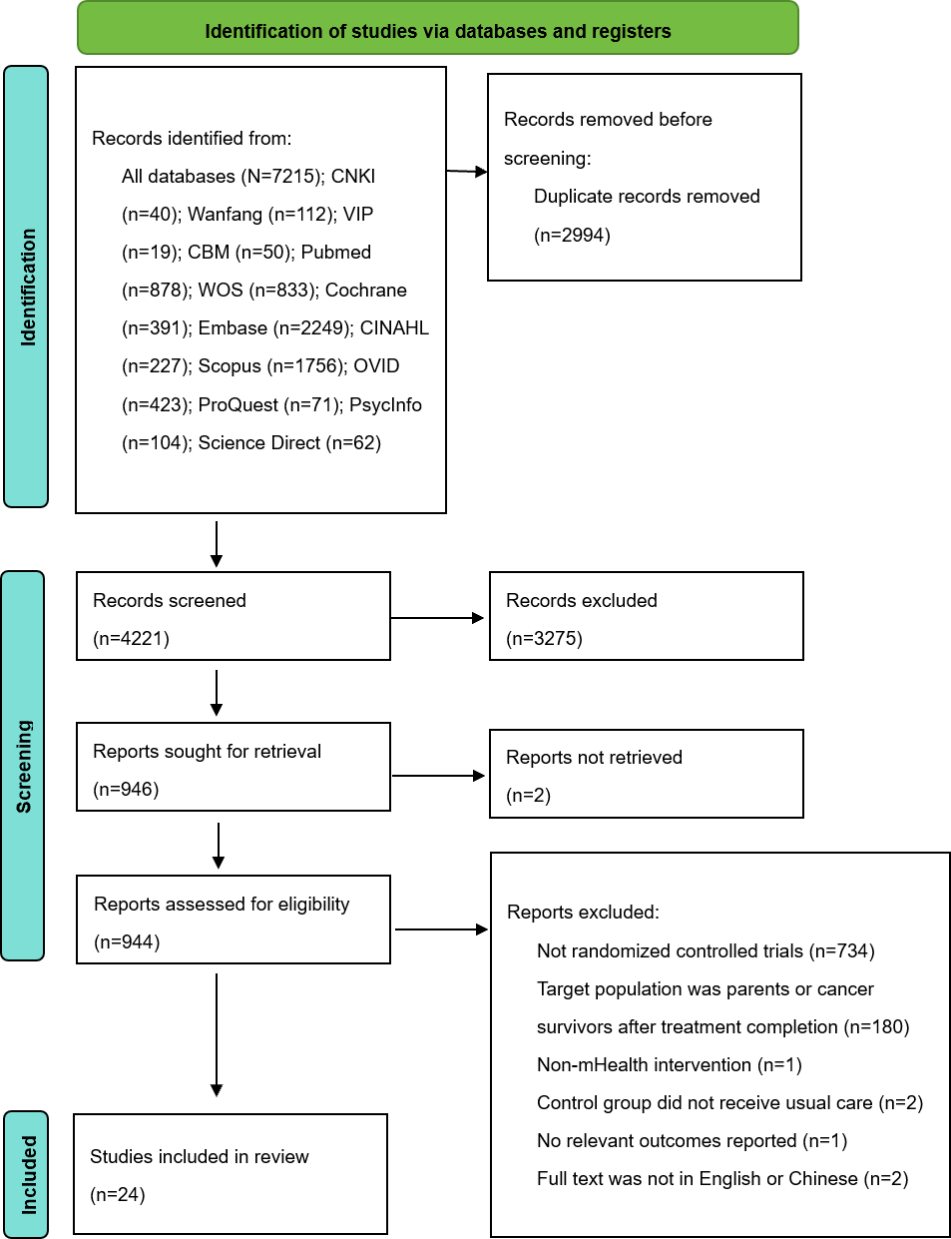
Study flowchart. mHealth: mobile health.

A total of 7215 studies were identified, with 24 meeting the inclusion criteria for the systematic review and meta-analysis (Figure 1).

### Descriptions of the Included Studies

This study included 24 RCTs published between 2017 and 2025, the main characteristics of which are summarized in Table 1. Most of the studies (19) were conducted in China [30-48], while one each was conducted in America [49], Canada [50], Iran [51], the Netherlands [52], and Turkey [53]. These studies involved a total of 2645 children with cancer, with sample sizes ranging from 18 to 444 participants. The cancer types included hematologic malignancies, brain tumors, bone tumors, neuroblastoma, sarcomas, and other solid tumors. Participants were aged 0‐18 years. The intervention durations varied from 7 days to 2 years. The intervention settings included home-based (9 studies [30-33,35,37,41,51,52]), hospital-based (3 studies [34,50,53]), and combined home and hospital (12 studies [36,38-40,42-49]).

**Table 1.** Characteristics of the included studies.

Author, year, country	Sample size (T^a^,C^b^), n	Age^c^ (y)	Male/ female (T,C)	Cancer type	Duration (wk)	Setting	mHealth platform	BCT^d^ clusters	Intervention control groups	Outcome measurement tool
Zhong et al [30], 2025, China	70, 71	T=4.73 (2.59); C=5.07 (3.08)	35/35, 45/26	Leukemia	12	Home	WeChat	Goals and planning^e^, feedback and monitoring^f^, social support^g^, natural consequences^h^, reward and threat^i^	Usual care^j^	SEAMS^k^; missed or incorrect medication rate; unplanned hospitalization rate; platform usage evaluation
Simon et al [52], 2024, Netherlands	79, 79	T=7.5 (5.1); C=7.5 (5.4)	41/38, 38/41	Hematologic malignancies, neurological tumor, solid tumors	4	Home	KLIK Pijnmonitor software	Feedback and monitoring, shaping knowledge^l^, natural consequences, social support	Usual care	NRS-11^m^; BPI-SF^n^; ET^o^; app acceptance; pain incidence
Shahri et al [51], 2024, Iran	33, 33	T=15.55 (1.85); C=15.21 (1.79)	18/15, 12/21	Leukemia, lymphoma, glioma, osteosarcoma	4	Home	Website + SMS text messaging	Shaping knowledge, social support, natural consequences	Usual care	Gastrointestinal symptom incidence; gastrointestinal symptom self-management score
Zhao et al [31], 2023, China	40, 40	T=8.25 (2.02); C=8.30 (1.68)	20/21, 25/14	Leukemia	4	Home	WeChat	Goals and planning, feedback and monitoring, shaping knowledge, social support, comparison of outcomes^p^, natural consequences	Usual care	PICC^q^-related complications; catheter care status
Lv et al [32], 2023, China	50, 49	T=8.45 (2.52); C=8.77 (2.64)	29/21, 26/23	Brain tumors	12	Home	WeChat	Social support, shaping knowledge, feedback and monitoring, reward and threat	Usual care	KAP^r^ of catheter care; PICC-related complications; PedsQL 4.0^s^
Hu et al [33], 2022, China	45, 44	T=7.32 (2.02); C=7.49 (2.01)	24/21, 24/20	Leukemia	12	Home	WeChat	Social support, shaping knowledge, reward and threat, feedback and monitoring	Usual care	SCAS-C^t^; ESCA^u^; PICC-related complications
Semerci et al [53], 2022, Turkey	26, 31	T and C: 8‐18	15/11, 17/14	Bone tumors, leukemia, lymphoma, brain tumors	1	Hospital	“5inD” software	Repetition and substitution^v^, feedback and monitoring	Usual care	ARINVc^w^; ARINVp^x^
Breakey et al [50], 2022, Canada	39, 42	T and C: 15.2 (1.7)	26/10, 17/23	Leukemia, solid tumors, lymphoma, brain tumors	12	Hospital	Website	Shaping knowledge, social support, feedback and monitoring, goals and planning	Usual care	Satisfaction questionnaire ; REDCap^y^ logs; website engagement; AWC^z^ and caregiver self-reports
Ren et al [34], 2021, China	118, 118	T and C: 0‐18	33/26, 37/22	Brain tumors	≥3	Hospital	Medical software + WeChat + Hospital system	Shaping knowledge, goals and planning, social support, feedback and monitoring, reward and threat, natural consequences	Usual care	PPUS-CC^aa^; RHDS^ab^
Xie [35], 2021, China	30, 30	T=5.94 (1.81); C=5.96 (1.76)	20/10, 18/12	Wilms tumor	8	Home	WeChat	Shaping knowledge, social support, goals and planning, feedback and monitoring	Usual care	SF-36^ac^; PSQI^ad^; Self-developed Treatment Adherence/ Satisfaction Scale
Jiang [36], 2021, China	40, 40	T=3.47 (1.27); C=3.52 (1.64)	23/17, 21/19	Leukemia	144	Hospital + Home	WeChat	Shaping knowledge, social support, goals and planning, repetition and substitution, regulation^ae^, natural consequences	Usual care	Self-developed Resistance/ Satisfaction Scale; CMFS^af^
Ye et al [37], 2021, China	9, 9	T=6.79 (1.15); C=6.83 (1.13)	5/4, 6/3	Leukemia	40	Home	WeChat	Shaping knowledge, social support	Usual care	Adherence to prescribed treatment
Li et al [38], 2020, China	30, 30	T=4.30 (1.75); C=4.28 (1.86)	23/17, 24/16	Malignant tumor	24	Hospital + Home	WeChat	Shaping knowledge, social support, goals and planning, feedback and monitoring	Usual care	PedsQL 4.0; PedsQL 3.0; nursing satisfaction questionnaire
Song et al [40], 2020, China	36, 36	T=4.39 (2.14); C=4.38 (2.13)	20/16, 21/15	Leukemia	—^ag^	Hospital + Home	WeChat＋Telephone	Shaping knowledge, social support, goals and planning, natural consequences	Usual care	PICC-related complications
Wang et al [39], 2020, China	30, 30	T and C: 0‐9	17/13, 18/12	Leukemia	48	Hospital + Home	WeChat	Shaping knowledge, social support, goals and planning, feedback and monitoring, comparison of outcomes, natural consequences	Usual care	Complication incidence; treatment adherence questionnaire
Yao et al [41], 2020, China	65, 65	T=11.40 (4.65); C=10.37 (3.97)	33/29, 35/25	Leukemia	12	Home	Medical software	Shaping knowledge, feedback and monitoring, social support, natural consequences	Usual care	PICC-related complications rate; catheter care omission rate; catheter maintenance time and cost; PICC-specific nursing service satisfaction scale; Children’s PICC Self-Management Ability Scale
Bhatia et al [49], 2020, United States	230, 214	T=8.6 (5.6‐14.3); C=7.5 (5.3‐14.0)	154/76, 148/66	Leukemia	20	Hospital + Home	Website + SMS text messaging + MEMS	Shaping knowledge, social support, repetition and substitution, feedback and monitoring, natural consequences	Usual care	MEMS^ah^ usage records to calculate adherence and safety
Huang et al [42], 2019, China	17, 15	T=6.5 (3.5); C=6.3 (3.7)	10/7, 7/8	Leukemia	144	Hospital + Home	WeChat	Social support, goals and planning, feedback and monitoring	Usual care	PedsQL 4.0; caregiver self-management ability score
Zhong [43], 2018, China	36, 36	T=6.3 (3.1); C=6.8 (2.6)	18/18, 20/16	Leukemia	3	Hospital + Home	WeChat	Shaping knowledge, social support, goals and planning, feedback and monitoring, natural consequences, social support, regulation	Usual care	PICC Knowledge Assessment Questionnaire; PICC Self-Management Ability Scale; PICC Patient Satisfaction Questionnaire; PICC-related complications rate
Wen [44], 2018, China	52, 50	T=8.8 (1.5); C=9.3 (1.8)	29/23, 26/24	Leukemia	12	Hospital + Home	WeChat	Shaping knowledge, social support, regulation, natural consequences	Usual care	HAD^ai^; PedsQL^TM^ 4.0; time of PICC catheterization and the incidence of complications
Lu et al [45], 2018, China	23, 23	T=5.1 (1.43); C=5.9 (1.85)	12/11, 13/10	Wilms tumor	96	Hospital + Home	WeChat	Shaping knowledge, feedback and monitoring, goals and planning, natural consequences	Usual care	Adherence to prescribed treatment; Quality of Life Scale
Ding et al [46], 2017, China	70, 70	—	—	Wilms tumor, neuroblastoma, hepatoblastoma, osteosarcoma	4	Hospital + Home	WeChat	Shaping knowledge, feedback and monitoring, social support, goals and planning, natural consequences	Usual care	PICC Knowledge Questionnaire; catheter care adherence; self-developed patient satisfaction questionnaire; PICC-related complications rate
Qin et al [47], 2017, China	70, 70	T=5.36 (1.58); C=5.62 (1.60)	41/29, 36/34	Lymphoma, rhabdomyosarcoma, neuroblastoma, Wilms tumor	48	Hospital + Home	WeChat	Shaping knowledge, social support, feedback and monitoring, comparison of outcomes, natural consequences	Usual care	PICC-related complications rate
Xiang et al [48], 2017, China	56, 56	T=5.3 (3.1); C=5.6 (3.5)	60, 52	Leukemia	—	Hospital + Home	WeChat + Telephone	Social support, feedback and monitoring, shaping knowledge, natural consequences	Usual care	PICC-related complications rate; self-developed patient satisfaction questionnaire

a T: experimental group.

b C: control group.

c Age is given as mean (SD), range values, and median (IQR).

d BCT: behavior change technique.

e *Goals and planning:* Personalized planning of medication, exercise, follow-up visits, disease management, and personal development.

f *Feedback and monitoring: *Recording medication information, disease management, assessments, and test results with data visualization, generating corresponding guidance and recommendations; providing regular reminders, alerts for critical values or risky behaviors; and enabling video consultations or scheduling in-person follow-ups.

g *Social support:* Establishing 2-way communication channels with other families and medical staff or offering assistance and reimbursement consultations.

h *Natural consequences:* Enabling individuals to understand or experience the natural consequences of their behaviors.

i *Reward and threat: *Using virtual tasks or gamified elements to assess the mastery of self-care knowledge and providing point-based rewards or badge recognition.

j *Usual care:* During hospitalization, treatments are administered according to medical orders, with face-to-face monitoring of vital signs and rehabilitation care, while maintaining a comfortable ward environment. Before discharge, guidance is provided via verbal instructions, printed materials, or health education videos, covering medication, diet, and catheter care. At home, care and record-keeping are performed by family members, with medical staff monitoring the condition and addressing questions through regular phone calls or outpatient follow-ups. This approach does not involve mobile apps or online platform support.

k SEAMS: Self-Efficacy for Appropriate Medication Use Scale.

l *Shaping knowledge:* Providing staged information delivery, search, and storage functions related to disease, symptoms, catheter care, and medication management.

m NRS-11: Numerical Rating Scale–11.

n BPI-SF: Brief Pain Inventory–short form.

o ET: emotion thermometer.

p *Comparison of outcomes:* Promoting belief enhancement and behavior change through social comparison and modeling by sharing successful cases, organizing parent communication for mutual learning, and demonstrating practical experiences and caregiving skills.

q PICC: peripherally inserted central catheter.

r KAP: knowledge, attitude, and practice.

s PedsQL 4.0: Pediatric Quality of Life Inventory 4.0.

t SCAS-C: Spence Children’s Anxiety Scale—child version.

u ESCA: Exercise of Self-Care Agency Scale.

v *Repetition and substitution:* Conducting online cognitive-behavioral interventions, professional psychological counseling, or other complementary medical interventions.

w ARINVc: Children’s Version of the Adapted Rhodes Index of Nausea and Vomiting.

x ARINVp: Parent Version of the Adapted Rhodes Index of Nausea and Vomiting.

y REDCap: Research Electronic Data Capture.

z AWC: adolescents with cancer.

aa PPUS-CC: Parent Perception of Uncertainty Scale–Child Chronic Illness version.

ab RHDS: Readiness for Hospital Discharge Scale.

ac SF-36: 36-Item Short Form Survey.

ad PSQI: Pittsburgh Sleep Quality Index.

ae *Regulation:* Helping individuals reduce negative emotions, relieve tension and stress, and maintain a more stable and positive psychological and physiological state through interventions such as stress management and music therapy.

af CMFS: Children’s Medical Fear Scale.

ag Not available.

ah MEMS: Medication Event Monitoring System.

ai HAD: Hospital Anxiety and Depression Scale.

**Figure 2.**
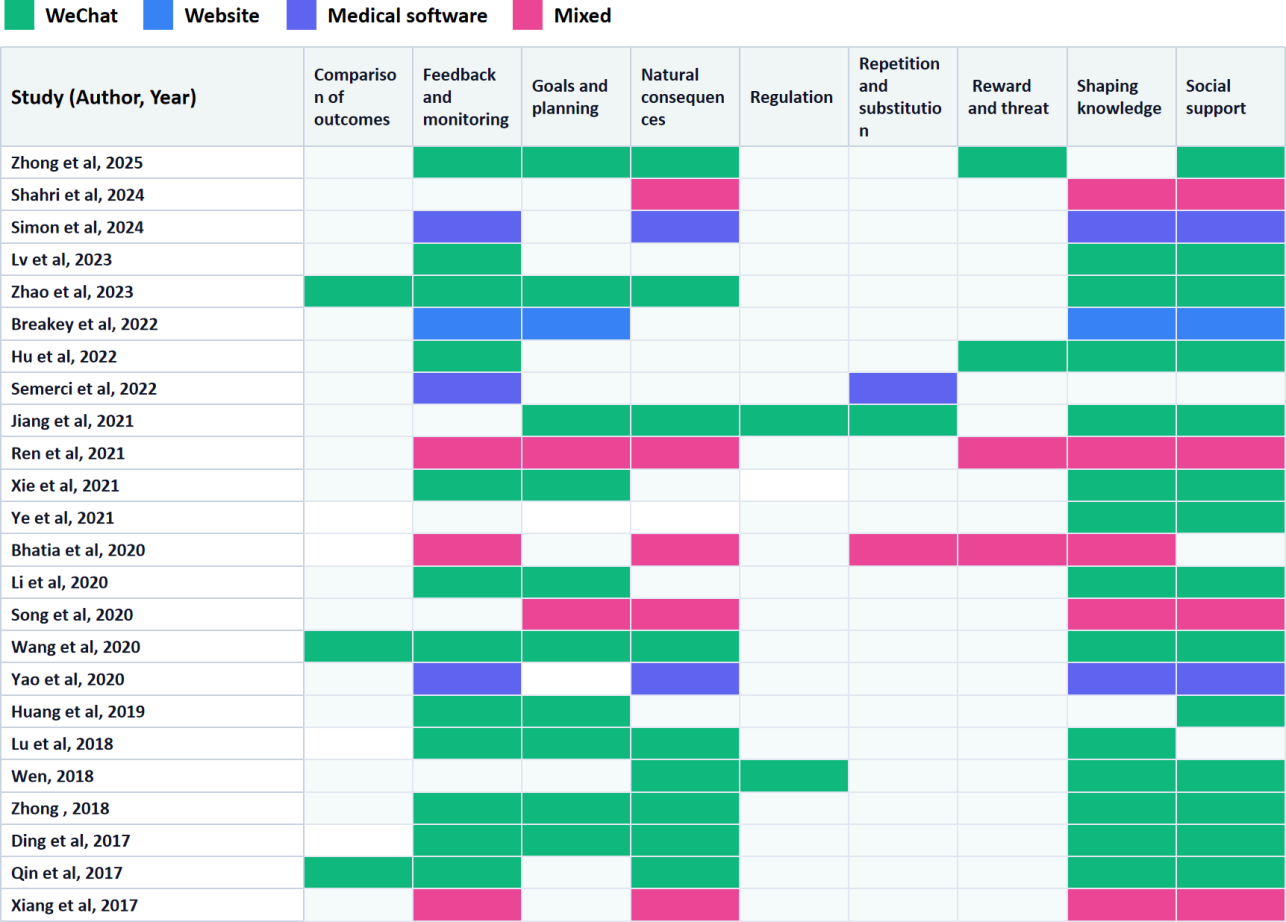
The behavior change techniques used in each study [30-53].

Regarding intervention platforms used for the experimental groups, 15 studies [30-33,35-39,42-47] used WeChat (a widely used mobile-based social media and communication app in China that supports messaging, group communication, and content dissemination[54]), 1 used a website [50], 3 used professional medical apps [41,52,53], and 5 used mixed platforms [34,40,48,49,51]. All interventions were conducted under the guidance of medical staff. Figure 2 shows the BCTs used in each study. A total of 13 studies involved goals and planning [30,31,34-36,38-40,42,43,45,46,50]; 19 studies included feedback and monitoring [30-35,38,39,41-43,45-50,52,53]; 21 provided social support [30-44,46-48,50-52]; 21 included shaping knowledge; 3 involved repetition and substitution [36,49,53]; 4 included reward and threat [30,32-34]; 3 conducted comparison of outcomes [31,39,47]; 16 incorporated natural consequences [30,31,34,36,39-41,43-49,51,52]; 2 involved regulation [36,44]. A total of 3 reported security measures such as password login and patient-specific data verification [41,50,51]. The control groups received usual care.

### Quality Assessment Results

**Figure 3.**
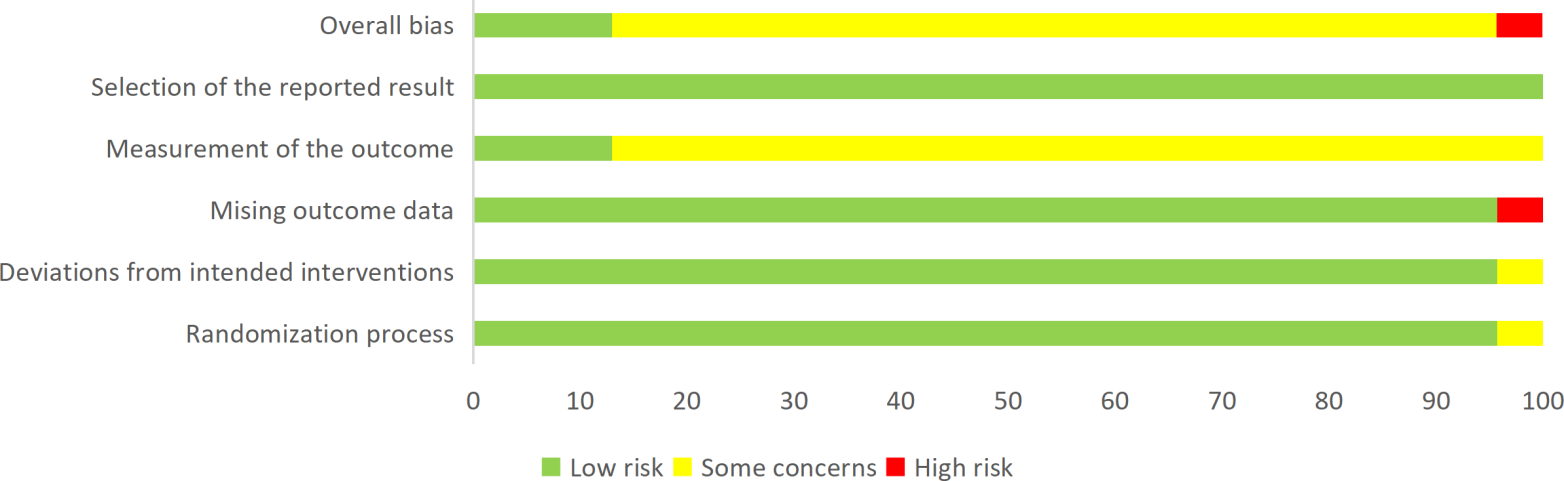
Risk of bias graph.

Among the 24 included studies (Figures3  4), 3 were rated as low risk of bias [32,41,49]. One study had a high risk due to unaddressed missing data [50]; the remaining 20 had some concerns (1 study [52] due to potential bias in the intervention, 1 study [37] for not reporting allocation concealment, and the other 18 due to subjective outcome assessments potentially influenced by positive psychological suggestion).

GRADE evidence quality ratings are presented in Multimedia Appendix 2. Evidence for infection incidence, overall incidence of PICC complications, PICC phlebitis, PICC puncture site bleeding, PICC puncture site infection, PICC occlusion, PICC catheter dislodgement, and PICC catheter displacement was rated as moderate. In contrast, evidence quality for QoL, PICC thrombogenesis, health knowledge, children’s self-management ability, and treatment adherence was rated as very low.

**Figure 4.**
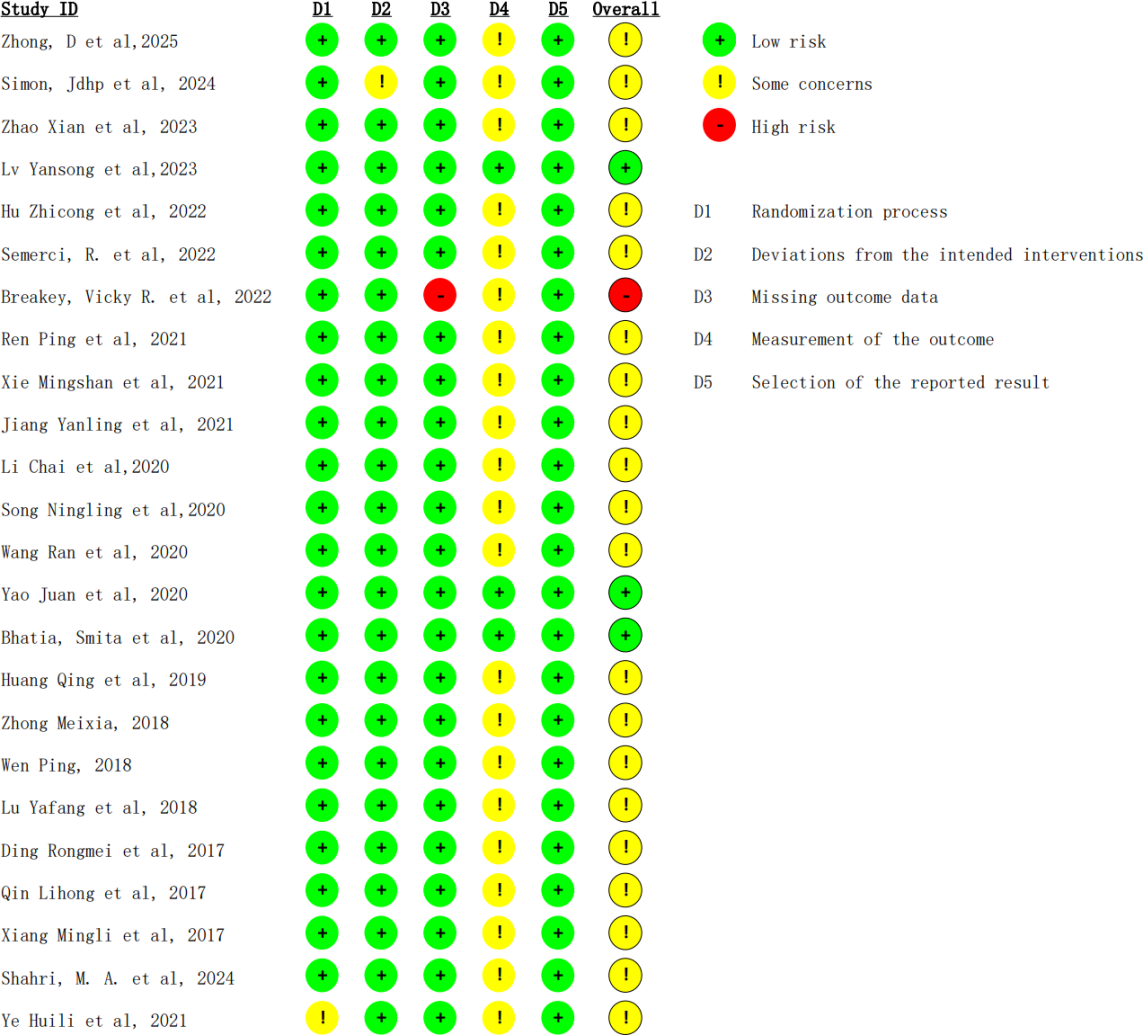
Risk of bias summary [30-53].

### Meta-Analysis Results

#### Infection Incidence

**Figure 5.**
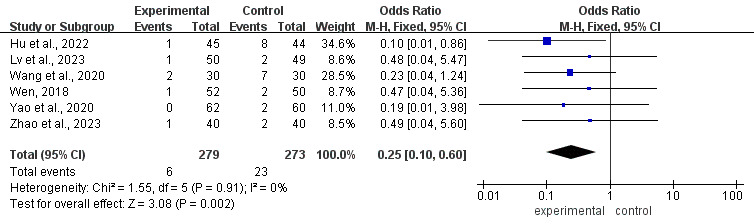
Forest plot of the effect of mHealth on infection incidence [31-33,39,41,44].

Six [31-33,39,41,44] of the 24 studies investigated the effectiveness of mHealth-based interventions in controlling infection rates among pediatric patients with cancer. The infections reported in the included studies primarily involved skin, bloodstream, catheter-related, oral, and perianal infections. The studies demonstrated homogeneity (*P*=.91; *I^2^*=0%), thus enabling analysis using a fixed-effects model. The results showed that mHealth-based interventions could significantly reduce the incidence of infections (OR 0.25, 95% CI 0.10-0.60; *P*=.002, Figure 5).

#### Quality of Life

**Figure 6.**
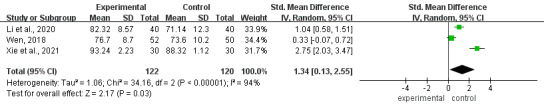
Forest plot of the effect of mobile health on quality of life [35,38,44].

Of the 24 studies included in the analysis, three [35,38,44] reported total QoL scale scores for pediatric patients with cancer who received mHealth-based interventions. Due to high heterogeneity across the studies (*P*<0.001; *I^2^*=94%), a random-effects model was used for the analysis. Meta-analysis results showed that mHealth-based interventions were more effective than control in improving the QoL of children with cancer (SMD =1.34, 95% CI 0.13-2.55; *P*=.03, Figure 6).

#### PICC-Related Complications

**Figure 7.**
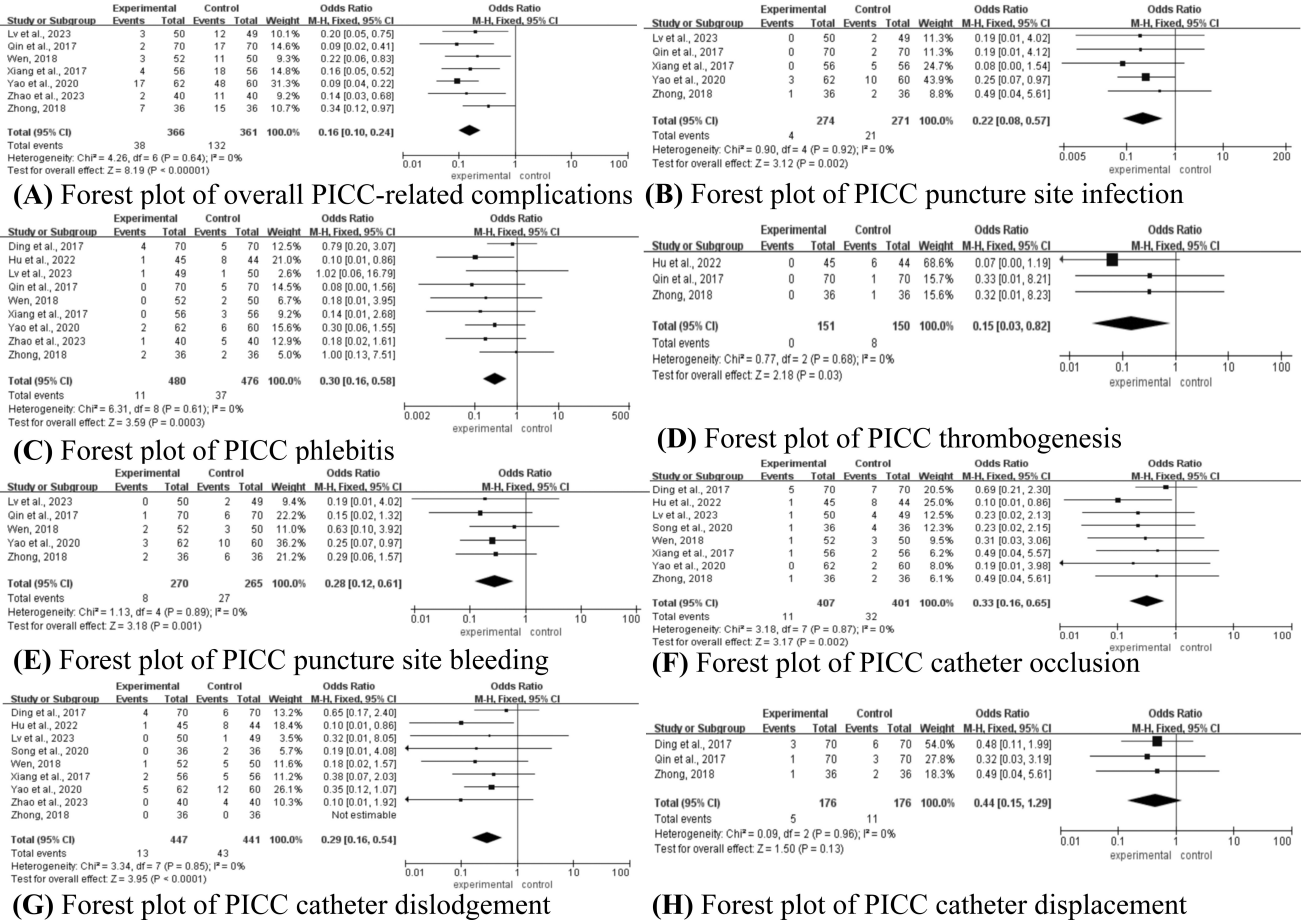
Forest plot of the effect of mHealth on (A) overall PICC-related complications [31,32,41,43,44,47,48], (B) PICC puncture site infection [32,41,43,47,48], (C) PICC phlebitis [31-33,41,43,44,46-48], (D) PICC thrombogenesis [33,43,47], (E) PICC puncture site bleeding [32,41,43,44,47], (F) PICC catheter occlusion [32,33,40,41,43,44,46,48], (G) PICC catheter dislodgement [31-33,40,41,43,44,46,48], and (H) PICC catheter displacement [43,46,47]. PICC: peripherally inserted central catheters.

Among the 24 studies, 10 [31-33,40,41,43,44,46-48] reported the incidence of PICC-related complications in mHealth interventions, including the overall incidence of PICC complications (n=7) [31,32,41,43,44,47,48], puncture site infection (n=5) [32,41,43,47,48], phlebitis (n=9) [31-33,41,43,44,46-48], thrombogenesis (n=3) [33,43,47], puncture site bleeding (n=5) [32,41,43,44,47], catheter occlusion (n=8) [32,33,40,41,43,44,46,48], catheter dislodgement (n=9) [31-33,40,41,43,44,46,48], and catheter displacement (n=3) [43,46,47]. Heterogeneity tests revealed no significant heterogeneity among studies for any outcome measure (*P*>.1; *I²*=0%), thus allowing analysis using a fixed-effect model. Meta-analysis results showed that in the mHealth intervention groups, the incidence of overall complications (OR 0.16, 95% CI 0.10-0.24; *P*<.001, Figure 7A), puncture site infection (OR 0.22, 95% CI 0.08-0.57; *P*=.002, Figure 7B), phlebitis (OR 0.30, 95% CI 0.16-0.58; *P*=.0003, Figure 7C), thrombogenesis (OR 0.15, 95% CI 0.03-0.82; *P*=.03, Figure 7D), puncture site bleeding (OR 0.28, 95% CI 0.12-0.61; *P*=.001, Figure 7E), catheter occlusion (OR 0.33, 95% CI 0.16-0.65; *P*=.002, Figure 7F) and catheter dislodgement (OR 0.29, 95% CI 0.16-0.54; *P*<.001, Figure 7G) were all lower than in the control groups. However, there was no significant difference in the incidence of catheter displacement (OR 0.44, 95% CI 0.15-1.29; *P*=.13, Figure 7H).

#### Health Knowledge

**Figure 8.**
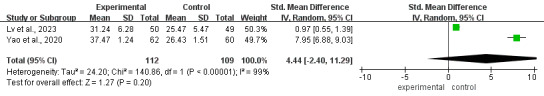
Forest plot of the effect of mobile health on health knowledge [32,41].

Among the 24 studies, 2 [32,41] reported the effects of mHealth-based interventions on health knowledge. Owing to substantial heterogeneity (*P*<.001; *I^2^*=99%), a random-effects model was used. There were no significant differences in health knowledge compared with the control group (SMD 4.44, 95% CI −2.40 to 11.29; *P*=.20, Figure 8).

#### Self-Management Ability

**Figure 9.**
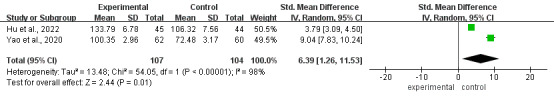
Forest plot of the effect of mobile health on self-management ability [33,41].

Among the 24 studies, 2 [33,41] reported the effects of mHealth-based interventions on self-management abilities in pediatric patients with cancer compared to usual care. Due to high heterogeneity across studies (*P*<.001; *I^2^*=98%), a random-effects model was used for analysis. The results of the meta-analysis indicated that mHealth-based interventions were more effective than control in improving children’s self-management abilities (SMD 6.39, 95% CI 1.26-11.53; *P*=.01, Figure 9).

#### Treatment Adherence

**Figure 10.**
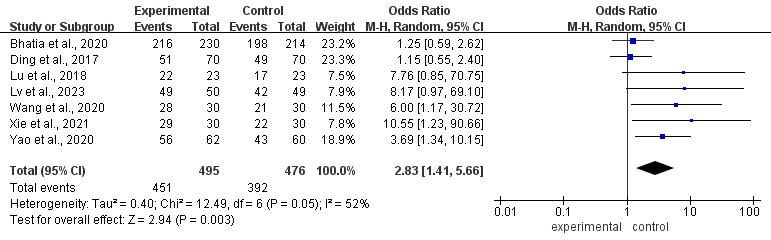
Forest plot of the effect of mobile health on treatment adherence [32,35,39,41,45,46,49].

Of the 24 studies, 10 [32,35-37,39,41,42,45,46,49] assessed the effectiveness of mHealth-based interventions compared to usual care in improving treatment adherence among pediatric patients with cancer. Two studies [36,42] were not pooled because of differences in scoring directions, and 1 study [37] was not entered into the quantitative synthesis owing to internally inconsistent and unverifiable adherence data; the remaining 7 studies reporting dichotomous adherence outcomes were included in the meta-analysis. Heterogeneity testing revealed significant heterogeneity among the studies (*P*=.05; *I^2^*=52%), which prompted the use of a random-effects model. The results indicated that children in the mHealth group had higher treatment adherence than those in the control group (OR 2.83, 95% CI 1.41-5.66; *P*=.003, Figure 10).

### Sensitivity Analyses

For most outcomes, excluding any single study did not alter the effect sizes of mHealth-based interventions, indicating stable results (see Multimedia Appendix 3). Exceptions were observed for QoL, PICC complication incidence, and treatment adherence. For quality of life, excluding either study by Li et al [38] or Xie [35] led to significant changes in the overall effect size (SMD 1.52, 95% CI −0.86 to 3.90; *P*=.21; SMD 0.67, 95% CI −0.03 to 1.38; *P*=.06), indicating instability. Exclusion study by Hu et al [33] (OR 0.33, 95% CI 0.03-3.19; *P*=.34), substantially altered the effect estimate for thrombogenesis. In contrast, heterogeneity in treatment adherence decreased markedly after excluding the study by Ding et al [46] (*P*=.11; *I^2^*=45%), while the pooled effect size remained stable (OR 3.72, 95% CI 1.68-8.22; *P*<.001). Two outcome indicators (health knowledge and self-management ability) were not subjected to sensitivity analysis as they were only involved in 2 studies.

### Publication Bias

The assessment of publication bias in the effects of mHealth–based interventions on infection incidence, QoL, PICC-related complications, health knowledge, self-management ability, and treatment adherence in pediatric patients with cancer was facilitated by the use of funnel plots. The majority of the funnel plots demonstrated symmetrical patterns in the outcomes, with the exception of those pertaining to health knowledge and self-management ability (Multimedia Appendix 4). In addition, the Egger test was performed to evaluate the incidence of infection (*t*=0.56; *P*=.61), the QoL (*t*=12.12; *P*=.052), PICC-related complications (*t*=0.42; *P*=.69), PICC puncture site infection (*t*=−0.66; *P*=.55), PICC phlebitis (*t*=−1.59; *P*=.16), PICC thrombogenesis (*t*=20.91; *P*=.03), PICC puncture site bleeding (*t*=−0.39; *P*=.72), PICC catheter occlusion (*t*=−2.35; *P*=.07), PICC catheter dislodgement (*t*=−2.09; *P*=.08), and PICC catheter displacement (*t*=−0.59; *P*=.66), and treatment adherence (*t*=5.37; *P*=.003). The results indicated the absence of substantial publication bias, with the exception of the PICC thrombogenesis, health knowledge, self-management ability, and treatment adherence.

### Subgroup Analysis and Meta-Regression

Subgroup analyses and meta-regression were not conducted because fewer than 10 studies were available for each outcome. This decision aligns with the Cochrane Handbook [55] guidelines to prevent false-positive results, insufficient statistical power, and unstable results.

## Discussion

### Principal Findings

This review aims to evaluate whether mHealth-based interventions are more effective than usual care for pediatric patients with cancer. The quality of the evidence for each outcome measure in the included studies was graded using GRADEpro, with the following results:

1. Moderate certainty evidence: mHealth-based interventions significantly reduce the incidence of infections and PICC complications (including overall complications, puncture site infection, phlebitis, puncture site bleeding, catheter occlusion, and catheter dislodgement) compared to usual care in pediatric patients with cancer, with no significant effect observed for PICC catheter displacement.
2. Very low certainty evidence: mHealth-based interventions outperform usual care in improving QoL, self-management ability, treatment adherence, and reducing the PICC thrombogenesis rate among pediatric patients with cancer, but do not significantly enhance their health knowledge.

### Effect of mHealth Interventions on Infection Rates

This is the first review to summarize the effect of mHealth on infection rates in pediatric patients with cancer, demonstrating a significant protective benefit. The interventions used 8 BCTs to establish effective bidirectional communication between clinicians and patients, offering practical insights for clinical implementation. Notably, the majority of interventions used WeChat, a social platform that dominates the Chinese digital landscape with over 1 billion monthly active users and preinstallation on more than 90% of smartphones across all age demographics. Its comprehensive functionality encompasses instant messaging (text, voice, and video messages), free audio and video calls, group chats (eg, interactions between health care professionals and patients or among patients), and private messaging for individualized consultation. In health care settings, WeChat is commonly used through official accounts (for health education dissemination), mini-programs (for data recording or questionnaire administration), and multimedia transmission (eg, image or video reports), offering advantages of low cost and wide reach [54]. Nevertheless, this app presents notable limitations. Interactions frequently depend on medical staff availability, potentially resulting in delayed responses [56]. In addition, current health education materials often require manual compilation and uploading, reducing efficiency and increasing clinician workload—potentially compromising care quality and safety. There is a clear need for more automated, dynamic content delivery systems.

### Effect of mHealth Interventions on QoL

The results of a meta-analysis indicate that mHealth-based interventions significantly improved the QoL for pediatric patients with cancer compared to usual care. These findings address previous gaps in quantitative measurement [15] and reconcile inconsistencies attributed to confounding factors in interventions and populations [14]. The included mHealth interventions incorporated 6 BCTs, supporting psychological, social, and symptom-related needs, thereby reducing risks of psychological distress, infections, and PICC complications. However, high heterogeneity and sensitivity analyses indicate instability in these results. All studies on this outcome measure used WeChat as the platform, differing only in sample size and measurement tools. This suggests that the instability and heterogeneity of these intervention outcomes may be influenced more by methodological factors than by mHealth. In addition, the observed large effect size of QoL may be partly attributable to the elevated effect size in Xie’s [35] study, which assessed outcomes at 2 months post intervention—a shorter timeframe than other studies (3 mo, 6 mo)—resulting in stronger short-term effects. Furthermore, the employment of subjective assessment scales in all studies may have resulted in an overestimation of results. Therefore, while mHealth interventions show potential for improving the QoL in pediatric patients with cancer, their long-term effects require further confirmation through more rigorous evidence-based studies before integration into routine clinical care.

### Effect of mHealth Interventions on Incidence of PICC-Related Complications

This study is the first to summarize the effectiveness of mHealth-based interventions in reducing PICC-related complications within the pediatric oncology field. Results indicate that mHealth management significantly lowers overall complication rates compared to usual care, likely by mitigating recall bias and misunderstandings associated with traditional education methods (eg, demonstrations or printed manuals) [57]. The mHealth intervention in this outcome involved 8 BCTs, offering real-time monitoring and correction, scheduling and reminders, online assessment, and follow-up. Despite these advantages, the nonsignificant effect of mHealth on reducing catheter displacement may be attributed to children’s growth and frequent postural changes, which increase the risk of mechanical traction [58]. Hu et al [33] implemented a reward and threat system (weekly quizzes with prizes for successful venous catheterization) that generated a more pronounced intervention advantage in the intervention group compared to the control group. In contrast, the remaining 2 studies [43,47] used health education manuals in their control groups, potentially reducing the distinction from the experimental group. This suggests that certain functions of mHealth may not necessarily offer a significant advantage over health education manuals for specific outcomes. Overall, mHealth appears effective for reducing PICC complications, though efficacy may vary by complication type and specific intervention components.

### Effect of mHealth Interventions on Treatment Adherence

Similar to previous reviews [15], treatment adherence was significantly better in the mHealth group than in the usual care group. The mHealth intervention in this study integrated 9 BCTs. These strategies collectively address core aspects of treatment adherence, such as timely medication administration, maintaining appropriate lifestyle habits, and attending regular follow-up appointments and assessments [59]. Additionally, dedicated online accounts facilitate peer interaction, reducing stigma and enhancing adherence [60]. However, these results exhibit some heterogeneity and extreme effect sizes. The observed heterogeneity may be partly attributable to the 3-arm randomized controlled design used by Ding et al [46]. The extreme effect sizes may be related to the smaller sample sizes. Notably, while mHealth interventions significantly improve adherence among pediatric patients with cancer, participant attrition remains a significant issue. This may stem from limited digital literacy among children and their parents, which can hinder the effective use of mobile devices and negatively impact adherence [61]. Therefore, clinical practice should establish timely feedback and reminder mechanisms, which may be key factors in maintaining long-term engagement among pediatric patients.

### Effect of mHealth Interventions on Health Knowledge and Self-Management Ability

This review found that mHealth-based interventions did not significantly improve health knowledge scores, possibly due to a lack of age-appropriate educational content. The 4 BCT clusters used may not suffice for children’s cognitive levels [62]. Future interventions should incorporate age-tailored content, interactive designs, and parental involvement to enhance learning outcomes. In contrast, mHealth interventions demonstrated favorable effects on improving children’s self-management ability. This result pertains to 5 BCT clusters. Benefits likely stem from flexible, real-time education and feedback [63], empowering both children and caregivers [62]. The high effect sizes may reflect the use of subjective scales and potential Hawthorne effects.

However, Yao et al [41] used a professional medical platform for intervention, yielding effect sizes higher than those reported in studies using WeChat platforms [32,33] for both outcome measures. This discrepancy may explain the higher heterogeneity and wider confidence intervals observed, warranting cautious interpretation of the findings. Notably, Yao et al [41] was also the only study to conduct a cost-related analysis, reporting that the cost of hospital visits for PICC maintenance in the intervention group 117.50 CNY (US $17.07; IQR 105.00-132.50) was significantly lower than that in the control group 321 CNY (US $46.66; IQR 261.50-374.25), limiting the generalizability of the evidence. Therefore, balancing patient economic benefits with clinician workload remains a key challenge for health care administrators.

### Strengths and Limitations

This review has several strengths. First, it provides a systematic review and meta-analysis of mHealth effectiveness in pediatric cancer, moving beyond usability to report quantitative clinical outcomes—including infection rates and PICC complications—for the first time. It also offers evidence on QoL, health knowledge, self-management, and treatment adherence, establishing a foundation for future research and practice. Second, by including only RCTs—the gold standard in clinical evidence—and restricting participants to pediatric patients undergoing treatment (excluding parents or survivors), it minimizes confounding and enhances translational potential. Third, it identifies and categorizes BCTs across studies, standardizing intervention components and improving comparability and replicability for future design. Fourth, the inclusion of Chinese databases supplements evidence from developing countries, addressing a gap in prior research.

Limitations should also be acknowledged. First, most included studies lacked the implementation of blinding, resulting in a current lack of high-certainty evidence. This may be related to the inherent difficulty of setting up blinding for mHealth interventions. Second, 79% of the included studies were conducted in China, with interventions predominantly delivered via the WeChat platform. However, the usage context of WeChat differs from that of mHealth tools commonly used in other countries, such as standalone mHealth apps or web-based platforms. The integration of daily life functions with health care services within WeChat may result in higher user engagement and retention compared with independent medical apps, potentially representing a key factor underlying the observed intervention effectiveness. Consequently, this WeChat-based model, characterized by high user engagement, may be difficult to directly replicate in other countries or regions with different health care systems and mHealth infrastructure, potentially limiting the generalizability of the findings to other linguistic and cultural contexts. Third, as a nonmedical social platform, WeChat has limitations in clinical applications. Specifically, it shows deficiencies in data encryption, regulatory compliance, liability definition, and ethical oversight during use. Simultaneously, medical staff may experience vigilance fatigue and blurred professional boundaries during specialized communications on such platforms due to unclear responsibility frameworks [64]. Moreover, it also lacks integration with clinical systems (eg, electronic health records), structured data support, and advanced algorithmic feedback [65]. Fourth, the limited number of included studies precluded subgroup analysis or meta-regression, restricting deeper exploration of heterogeneity sources, including the influence of BCT number on outcomes. Fifth, only Chinese and English articles were included due to the authors’ reading limitations, which may have resulted in the omission of high-quality articles in other languages. Finally, although the initial protocol registration planned to include a range of telehealth interventions and study designs, the scope of the review was refined during the implementation phase to focus specifically on RCTs of mHealth-based interventions. This refinement improved conceptual clarity and internal validity, while also strengthening the clinical and practical relevance of the findings. However, this focused scope may limit the applicability of the conclusions to other forms of telehealth (such as telephone-based consultations or video-enabled remote care) and to nonrandomized study designs that more closely reflect real-world practice (eg, stepped-wedge or cluster-based designs).

### Prospects

The findings of this study suggest that the extant evidence remains inadequate, and subsequent research may investigate the following domains.

First, future platforms could integrate ecological momentary assessment and smart biosensors to enable dynamic, predictive monitoring. Establishing automated, risk-stratified feedback mechanisms and exploring the incorporation of large language models could enhance efficiency through automated content updates and personalized information delivery. This would allow clinicians to focus more on data review and clinical decision-making [66]. Second, given the advantage of WeChat highlighted in this review, future developments should prioritize embedding mHealth modules into widely used ecosystem apps to minimize user burden, rather than developing isolated apps. Third, research quality should be enhanced. Adopting internationally recognized core outcome sets would facilitate cross-study comparisons [67]. Large-scale, multicenter RCTs should be conducted with extended follow-up periods to observe the temporal dynamics of intervention effects and long-term clinical outcomes. Fourth, equity and safety should be prioritized. Preuse assessments of digital health literacy and preferences among patients, parents, and health care providers should be conducted, offering tailored training to ensure resource equity during mHealth implementation. Platform users should be clearly defined to mitigate the adverse effects of inappropriate mobile device use on children’s vision, sleep, and other areas [68]. Finally, implementation research should be strengthened. Clear cost-benefit analyses should be conducted; barriers and facilitators to mHealth adoption should be evaluated, such as health care institutions’ feasibility regarding equipment investment, technical maintenance, staffing costs, and time allocation; and how mHealth interventions integrate with existing care processes and electronic information systems should be examined to avoid additional workload, duplicate documentation, or process conflicts, thereby enhancing technology usability and sustainability.

### Conclusion

This systematic review and meta-analysis synthesizes evidence from RCTs on mHealth interventions for children with cancer. The findings suggest that mHealth holds significant value in pediatric oncology care, demonstrating greater effectiveness than usual care in reducing the incidence of infection and various PICC-related complications, including overall complications, site infection, phlebitis, thrombosis, bleeding, occlusion, and dislodgement. mHealth interventions also significantly improved patients’ QoL, self-management ability, and treatment adherence. However, current evidence does not show a significant advantage for mHealth in reducing PICC catheter displacement or enhancing health knowledge. Large effect sizes for some outcomes may be related to subjective measurement tools and small sample sizes. Limitations such as lack of blinding and considerable heterogeneity reduce the certainty of evidence and may lead to overestimation of effects; therefore, results should be interpreted cautiously. Despite these limitations, existing evidence supports the potential benefits and applicability of mHealth in pediatric cancer care. Future research should conduct higher-quality, multinational RCTs and use methods such as network meta-analysis to identify the most effective BCTs within mHealth platforms. Concurrently, integrating implementation science and cost-effectiveness analyses will be crucial, with a focus on aligning mHealth technologies with existing clinical workflows to improve their feasibility, scalability, and sustainable impact.

## Supplementary material

10.2196/86836 Multimedia Appendix 1 Search strategy.

10.2196/86836 Multimedia Appendix 2 GRADE assessment results.

10.2196/86836 Multimedia Appendix 3 Results of sensitivity analyses.

10.2196/86836 Multimedia Appendix 4 Funnel plots.

10.2196/86836 Checklist 1 PRISMA checklist.

## Abbreviations

BCT: behavior change technique
GRADE: Grading of Recommendations Assessment, Development, and Evaluation
IQR: Interquartile Range
MeSH: Medical Subject Headings
mHealth: mobile health
OR: odds ratios
PICC: peripherally inserted central catheter
PRISMA: Preferred Reporting Items for Systematic Reviews and Meta-Analyses
QoL: quality of life
RCT: randomized controlled trial
SMD: standardized mean difference
