# Supplementary material for: Effectiveness of Mobile Health Interventions in Pediatric Cancer: Systematic Review and Meta-Analysis of Randomized Controlled Trials
Source: JMIR Mhealth Uhealth. 2026 Apr 22;14:e86836. doi: 10.2196/86836 (PMC13102325; doi:10.2196/86836)
Supplement: Multimedia Appendix 1 [file mhealth-v14-e86836-s001.docx]

**Search Strategy**

| **Database** | **Search Strategy** | **Results** |
| --- | --- | --- |
| **CNKI** | (主题:“患儿”+“儿童”+“小儿”) AND (主题:“肿瘤”+“癌症”+“癌”白血病”+“淋巴瘤”+“恶性”+“霍奇金病”) AND (主题:“移动医疗”+“远程医疗”+“移动健康”+“远程健康”+“远程会诊”+“电子健康”+“应用程序”+“app”+“微信”) | 40 |
| **Wanfang** | (((患儿 OR 儿童 OR 小儿） AND (肿瘤 OR 癌症 OR 癌 OR 白血病 OR 淋巴瘤 OR 恶性 OR 霍奇金病)) AND (移动医疗 OR 远程医疗 OR 移动健康 OR 远程健康 OR 远程会诊 OR 电子健康 OR 应用程序 OR app OR 微信) | 112 |
| **VIP** | M=(肿瘤 OR 癌症 OR 癌 OR 白血病 OR 淋巴瘤 OR 恶性 OR 霍奇金病) AND M=(儿童 OR 患儿 OR 小儿) AND M=(移动医疗 OR 远程医疗 OR 移动健康 OR 远程健康 OR 远程会诊 OR 电子健康 OR 应用程序 OR app OR 微信) | 19 |
| **CBM** | #1 "儿童"[不加权：扩展]  #2 "肿瘤"[不加权：扩展]  #3 "远程医学"[不加权：扩展]  #4 "患儿"[常用字段：智能] OR "儿童"[常用字段：智能] OR "小儿"[常用字段：智能]  #5 (#4) OR (#1)  #6 "肿瘤"[常用字段：智能] OR "癌症"[常用字段：智能] OR "癌"[常用字段：智能] OR "白血病"[常用字段：智能] OR "淋巴瘤"[常用字段：智能] OR "恶性"[常用字段：智能] OR "霍奇金病"[常用字段：智能]  #7 (#6) OR (#2)  #8 "移动医疗"[常用字段：智能] OR "远程医疗"[常用字段：智能] OR "移动健康"[常用字段：智能] OR "远程健康"[常用字段：智能] OR "远程会诊"[常用字段：智能] OR "电子健康"[常用字段：智能] OR "应用程序"[常用字段：智能] OR "app"[常用字段：智能] OR "微信"[常用字段：智能]  #9 (#8) OR (#3)  #10 (#9) AND (#7) AND (#5)  12) (#3) AND (#6) AND (#11) | 50 |
| **PubMed** | #1 (Text Messaging[MeSH Terms]) OR (Internet - Based Intervention[MeSH Terms]) OR (Telemedicine[MeSH Terms]) OR (Telenursing[MeSH Terms]) OR (Smartphone[MeSH Terms]) OR (MobileHealth[Title/Abstract]) OR (mHealth[Title/Abstract]) OR (Telehealth[Title/Abstract]) OR (Telemetries[Title/Abstract]) OR (Smartphone[Title/Abstract]) OR (Software Application*[Title/Abstract]) OR (Mobile Application*[Title/Abstract]) OR (ElectronicApplication*[Title/Abstract]) OR (App[Title/Abstract]) OR (WeChat[Title/Abstract])  #2 (Child[MeSH Terms]) OR (Adolescent[MeSH Terms]) OR (Child*[Title/Abstract]) OR (Pediatric[Title/Abstract]) OR (adolescent*[Title/Abstract]) OR (Teen*[Title/Abstract]) OR (Youth*[Title/Abstract])  #3 (cancer*[Title/Abstract]) OR (oncology[Title/Abstract]) OR (tumor*[Title/Abstract]) OR (Neoplas*[Title/Abstract]) OR (Neoplasms[MeSH Terms]) OR (Malignan*[Title/Abstract]) OR (Leukemia[Title/Abstract]) OR (Leucocythemia[Title/Abstract]) OR (leukemia[MeSH Terms]) OR (lymphoma[Title/Abstract]) OR (neuroblastoma[Title/Abstract]) OR (A*sarcoma[Title/Abstract]) OR (Astrocytoma[Title/Abstract])  #4 #1 AND #2 AND #3 | 878 |
| **Web of Science** | ((TS=(cancer* OR Oncology OR tumor* OR Neoplas* OR Neoplasms OR Malignan* OR Leuk*emia* OR Leucocyth*emia* OR leukemia OR Lymphoma OR Neuroblastoma OR A*sarcoma OR Astrocytoma)) AND TS=(Child* OR Pediatric OR adolescen* OR Teen* OR Youth*)) AND TS=(Text Messaging OR Internet-Based Intervention OR Telemedicine OR Telenursing OR Smartphone OR MobileHealth OR mHealth OR Telehealth OR Telemetries OR Smart*phone* OR Software Application* OR Mobile Application* OR ElectronicApplication* OR App OR WeChat) | 833 |
| **Cochrane** | #1 MeSH descriptor: [Neoplasms] explode all trees  #2 MeSH descriptor: [Leukemia] explode all trees 6676  #3 (cancer*):ti,ab,kw OR (Oncology):ti,ab,kw OR (tumor*):ti,ab,kw OR (Neoplas*):ti,ab,kw OR (Malignan*):ti,ab,kw OR (Leuk*emia*):ti,ab,kw OR (Leucocyth*emia*):ti,ab,kw OR (Lymphoma):ti,ab,kw OR (Neuroblastoma):ti,ab,kw OR (A*sarcoma):ti,ab,kw OR (Astrocytoma):ti,ab,kw  #4 #1 OR #2 OR #3  #5 MeSH descriptor: [Child] explode all trees  #6 MeSH descriptor: [Adolescent] explode all trees  #7 MeSH descriptor: [Pediatrics] explode all trees  #8 (Child*):ti,ab,kw OR (adolescen*):ti,ab,kw OR (Teen*):ti,ab,kw OR (Youth*):ti,ab,kw  #9 #5 OR #6 OR #7 OR #8  #10 MeSH descriptor: [Text Messaging] explode all trees  #11 MeSH descriptor: [Internet-Based Intervention] explode all trees  #12 MeSH descriptor: [Telemedicine] explode all trees  #13 MeSH descriptor: [Telenursing] explode all trees  #14 MeSH descriptor: [Smartphone] explode all trees  #15 (MobileHealth):ti,ab,kw OR (mHealth):ti,ab,kw OR (Telehealth):ti,ab,kw OR (Telemetries):ti,ab,kw OR (Smart*phone*):ti,ab,kw OR (Software Application*):ti,ab,kw OR (Mobile Application*):ti,ab,kw OR (ElectronicApplication*):ti,ab,kw OR (App):ti,ab,kw OR (WeChat):ti,ab,kw  #16 #10 OR #11 OR #12 OR #13 OR #14 OR #15 35782  #17 #4 AND #9 AND #16 | 391 |
| **EMBASE** | #1 'neoplasm'/exp  #2 'leukemia'/exp  #3 'cancer*':ab,ti OR 'oncology':ab,ti OR 'tumor*':ab,ti OR 'neoplas*':ab,ti OR 'malignan*':ab,ti OR 'leukemia':ab,ti OR 'leucocythemia':ab,ti OR 'lymphoma':ab,ti OR 'neuroblastoma':ab,ti OR 'A*sarcoma':ab,ti OR 'Astrocytoma':ab,ti  #4 #1 OR #2 OR #3  #5 'child'/exp  #6 'pediatric'/exp  #7 'adolescent'/exp  #8 'pediatric':ab,ti OR 'child':ab,ti OR 'adolescen*':ab,ti OR 'adolescent':ab,ti OR 'teen*':ab,ti OR 'youth*':ab,ti  #9: #5 OR #6 OR #7 OR #8  #10 'text messaging'/exp  #11 'web-based intervention'/exp  #12 'telemedicine'/exp  #13 'telenursing'/exp  #14 'smartphone'/exp  #15 'text messaging':ab,ti OR 'internet-based intervention':ab,ti OR 'telemedicine':ab,ti OR 'telenursing':ab,ti OR 'smartphone':ab,ti OR 'mobilehealth':ab,ti OR 'mhealth':ab,ti OR 'telehealth':ab,ti OR 'telemetries':ab,ti OR 'smartphone':ab,ti OR 'software application*':ab,ti OR 'mobile application*':ab,ti OR 'electronicapplication*':ab,ti OR 'app':ab,ti OR 'wechat':ab,ti  #16 #10 OR #11 OR #12 OR #13 OR #14 OR #15  #17 #4 AND #9 AND #16 | 2249 |
| **CINAHL** | S1: XB (cancer OR Oncology OR tumor OR Neoplas OR Neoplasms OR Malignan OR Leukemia OR Leucocythemia OR Lymphoma OR Neuroblastoma OR Asarcoma OR Astrocytoma) OR MH Neoplasms OR MH leukemia  S2: [XB (Child OR Pediatric OR Adolescent OR Teen OR Youth) OR MH Pediatric OR MH Child OR MH Adolescent](https://webvpn.uestc.edu.cn/https/77726476706e69737468656265737421e2f2529926226b58300d8bbf9b5a6d36e960/search/results?db=ccm&expanders=concept&limiters=None&q=XB%20(Child%20OR%20Pediatric%20OR%20Adolescent%20OR%20Teen%20OR%20Youth)%20OR%20MH%20Pediatric%20OR%20MH%20Child%20OR%20MH%20Adolescent&qm=W3sidmFsdWUiOiJDaGlsZCBPUiBQZWRpYXRyaWMgT1IgQWRvbGVzY2VudCBPUiBUZWVuIE9SIFlvdXRoIiwidHlwZSI6ImZpZWxkIiwiY29kZSI6IlhCIn0seyJ2YWx1ZSI6Ik9SIiwidHlwZSI6ImxvZ2ljIn0seyJ2YWx1ZSI6IlBlZGlhdHJpYyIsInR5cGUiOiJmaWVsZCIsImNvZGUiOiJNSCJ9LHsidmFsdWUiOiJPUiIsInR5cGUiOiJsb2dpYyJ9LHsidmFsdWUiOiJDaGlsZCIsInR5cGUiOiJmaWVsZCIsImNvZGUiOiJNSCJ9LHsidmFsdWUiOiJPUiIsInR5cGUiOiJsb2dpYyJ9LHsidmFsdWUiOiJBZG9sZXNjZW50IiwidHlwZSI6ImZpZWxkIiwiY29kZSI6Ik1IIn1d&searchMode=boolean&sort=relevance&userDirectAction=true&isDashboardExpanded=true)  S3: XB (Text Messaging OR Internet-Based Intervention OR Telemedicine OR Telenursing OR Smartphone OR MobileHealth OR mHealth OR Telehealth OR Telemetries OR Mobile Application) OR MH Text Messaging OR MH Internet-Based Intervention OR MH Telemedicine OR MH Telenursing OR MH Smartphone  S6:S1 AND S2 AND S3 | 99 |
| **Scopus** | ( TITLE-ABS-KEY ("oncology" OR "Tumor" OR "cancer" ) AND TITLE-ABS-KEY ("child" OR "adolescent" OR "teenager" ) AND TITLE-ABS-KEY ("mHealth" OR "MobileHealth" OR "Telehealth") | 1756 |
| **Science Direct** | ("oncology" OR "Tumor" OR "cancer") AND ("child" OR "adolescent" OR "teenager") AND ("mHealth" OR "MobileHealth" OR "Telehealth") | 62 |
| **OVID** | #1: (Cancer* or Oncology or tumor* or Neoplas* or Neoplasms or Malignan* or Leukemia or Leucocythemia or leukemia or Lymphoma or Neuroblastoma or Asarcoma or Astrocytoma).ab. or (Cancer or Oncology or tumor* or Neoplas* or Neoplasms or Malignan* or Leukemia or Leucocythemia or leukemia or Lymphoma or Neuroblastoma or Asarcoma or Astrocytoma).ti.  #2: (Child or Pediatric or Child or adolescen* or Adolescent or Teen* or Youth*).ab. or (Child* or Pediatric or Child or adolescen* or Adolescent or Teen* or Youth*).ti.  #3: (Text Messaging or Internet-Based intervention or Telemedicine or Telenursing or Smartphone or MobileHealth or mHealth or Telehealth or Telemetries or Smartphone or Software Application* or Mobile Application* or ElectronicApplication* or App or WeChat).ab. or (Text Messaging or Internet-Based intervention or Telemedicine or Telenursing or Smartphone or MobileHealth or mHealth or Telehealth or Telemetries or Smartphone or Software Application* or Mobile Application* or ElectronicApplication* or App or WeChat).ti.  #4: #1 AND #2 AND #3 | 423 |
| **ProQuest** | S1:mainsubiect("oncology" OR "Tumor" OR "cancer") OR title("oncology" OR "Tumor" OR "cancer") OR abstract("oncology" OR "Tumor" OR "cancer")  S2:mainsubject("child" OR "adolescent" OR "teenager") OR title("child" OR "adolescent" OR "teenager") OR abstract("child" OR "adolescent" OR "teenager")  S3:mainsubiect("mHealth" OR "MobileHealth" OR "Telehealth") OR title("mHealth" OR "MobileHealth" OR "Telehealth") OR abstract("mHealth" OR "MobileHealth" OR "Telehealth")  S4:[S1] AND [S2] AND [S3] | 389 |
| **PsycInfo** | S1: Tl (Text Messaging OR Internet-Based Intervention OR Telemedicine OR Telenursing OR Smartphone OR MobileHealth OR mHealth OR Telehealth OR Telemetries OR Smartphone OR Software Application* OR Mobile Application* OR ElectronicApplication* OR App OR WeChat) OR AB (Text Messaging OR Internet-Based Intervention OR Telemedicine OR Telenursing OR Smartphone OR MobileHealth OR mHealth OR Telehealth OR Telemetries OR Smartphone OR Software Application* OR Mobile Application* OR ElectronicApplication* OR App OR WeChat)  S2: Tl (Child* OR Pediatric OR Child OR adolescen* OR Adolescent OR Teen* OR Youth*) OR AB (Child* OR Pediatric OR Child OR adolescen* OR Adolescent OR Teen* OR Youth*)  S3: Tl (cancer* OR Oncology OR tumor* OR Neoplas* OR Neoplasms OR Malignan* OR Leukemia OR Leucocythemia OR leukemia OR Lymphoma OR Neuroblastoma OR Asarcoma OR Astrocytoma) OR AB (cancer OR Oncology OR tumor* OR Neoplas* OR Neoplasms OR Malignan* OR Leukemia OR Leucocythemia OR leukemia OR Lymphoma OR Neuroblastoma OR A*sarcoma OR Astrocytoma)  S4: S1 AND S2 AND S3 | 104 |
| **Total** |  | 7215 |
