# Supplementary material for: Effectiveness of Mobile Health Interventions in Pediatric Cancer: Systematic Review and Meta-Analysis of Randomized Controlled Trials
Source: JMIR Mhealth Uhealth. 2026 Apr 22;14:e86836. doi: 10.2196/86836 (PMC13102325; doi:10.2196/86836)
Supplement: Multimedia Appendix 2 [file mhealth-v14-e86836-s002.docx]

**GRADE assessment results**

**Author(s):**

**Question:**

**Setting:**

**Bibliography:**

| **Certainty assessment** | | | | | | | **№ of patients** | | **Effect** | | **Certainty** | **Importance** |
| --- | --- | --- | --- | --- | --- | --- | --- | --- | --- | --- | --- | --- |
| **№ of studies** | **Study design** | **Risk of bias** | **Inconsistency** | **Indirectness** | **Imprecision** | **Other considerations** | **mhealth** | **control group** | **Relative (95% CI)** | **Absolute (95% CI)** |  |  |
| **Quality of Life (Scale from: 0.13 to 2.55)** | | | | | | | | | | | | |
| 3 | randomised trials | serious^a^ | very serious^b^ | not serious | very serious^c^ | none | 122 | 120 | - | SMD **1.34 SD higher** (0.13 higher to 2.55 higher) | ⨁◯◯◯ Very low^a,b,c^ |  |
| **Treatment Adherence** | | | | | | | | | | | | |
| 7 | randomised trials | serious^a^ | serious^b^ | not serious | not serious | publication bias strongly suspected^d^ | 451/495 (91.1%) | 392/476 (82.4%) | **OR 2.83** (1.41 to 5.66) | **106 more per 1,000** (from 45 more to 140 more) | ⨁◯◯◯ Very low^a,b,d^ |  |
| **Infection Incidence** | | | | | | | | | | | | |
| 6 | randomised trials | serious^a^ | not serious | not serious | not serious | none | 6/279 (2.2%) | 23/273 (8.3%) | **OR 0.25** (0.10 to 0.60) | **62 fewer per 1,000** (from 75 fewer to 32 fewer) | ⨁⨁⨁◯ Moderate^a^ |  |
| **Phlebitis** | | | | | | | | | | | | |
| 9 | randomised trials | serious^a^ | not serious | not serious | not serious | none | 11/480 (2.3%) | 37/476 (7.8%) | **OR 0.30** (0.16 to 0.58) | **53 fewer per 1,000** (from 64 fewer to 31 fewer) | ⨁⨁⨁◯ Moderate^a^ |  |
| **Puncture site bleeding** | | | | | | | | | | | | |
| 5 | randomised trials | serious^a^ | not serious | not serious | not serious | none | 8/270 (3.0%) | 27/265 (10.2%) | **OR 0.28** (0.12 to 0.61) | **71 fewer per 1,000** (from 88 fewer to 37 fewer) | ⨁⨁⨁◯ Moderate^a^ |  |
| **Catheter occlusion** | | | | | | | | | | | | |
| 8 | randomised trials | serious^a^ | not serious | not serious | not serious | none | 11/407 (2.7%) | 32/401 (8.0%) | **OR 0.33** (0.16 to 0.65) | **52 fewer per 1,000** (from 66 fewer to 26 fewer) | ⨁⨁⨁◯ Moderate^a^ |  |
| **Catheter dislodgement** | | | | | | | | | | | | |
| 9 | randomised trials | serious^a^ | not serious | not serious | not serious | none | 13/447 (2.9%) | 43/441 (9.8%) | **OR 0.29** (0.16 to 0.54) | **67 fewer per 1,000** (from 81 fewer to 42 fewer) | ⨁⨁⨁◯ Moderate^a^ |  |
| **PICC-Related Complications-Overall incidence** | | | | | | | | | | | | |
| 7 | randomised trials | serious^a^ | not serious | not serious | not serious | none | 38/336 (10.4%) | 132/361 (36.6%) | **OR 0.16** (0.10 to 0.24) | **281 fewer per 1,000** (from 311 fewer to 244 fewer) | ⨁⨁⨁◯ Moderate^a^ |  |
| **Self-Management ability** | | | | | | | | | | | | |
| 2 | randomised trials | serious | serious^b^ | not serious | serious^e^ | publication bias strongly suspected^d^ | 107 | 104 | - | SMD **6.39 higher** (1.26 higher to 11.53 higher) | ⨁◯◯◯ Very low^b,d,e^ |  |
| **Health knowledge** | | | | | | | | | | | | |
| 2 | randomised trials | not serious | serious^b^ | not serious | serious^f^ | publication bias strongly suspected^d^ | 112 | 109 | - | SMD **4.44 SD higher** (2.4 lower to 11.29 higher) | ⨁◯◯◯ Very low^b,d,f^ |  |
| **Puncture site infection** | | | | | | | | | | | | |
| 5 | randomised trials | serious^a^ | not serious | not serious | not serious | none | 4/274 (1.5%) | 21/271 (7.7%) | **OR 0.22** (0.08 to 0.57) | **59 fewer per 1,000** (from 71 fewer to 32 fewer) | ⨁⨁⨁◯ Moderate^a^ |  |
| **Thrombogenesis** | | | | | | | | | | | | |
| 3 | randomised trials | serious^a^ | not serious | not serious | serious^c^ | publication bias strongly suspected^d^ | 0/151 (0.0%) | 8/150 (5.3%) | **OR 0.15** (0.03 to 0.82) | **45 fewer per 1,000** (from 52 fewer to 9 fewer) | ⨁◯◯◯ Very low^a,c,d^ |  |
| **Catheter displacement** | | | | | | | | | | | | |
| 3 | randomised trials | serious^a^ | not serious | not serious | not serious | none | 5/176 (2.8%) | 11/176 (6.3%) | **OR 0.44** (0.15 to 1.29) | **34 fewer per 1,000** (from 53 fewer to 17 more) | ⨁⨁⨁◯ Moderate^a^ |  |

**CI:** confidence interval; **OR:** odds ratio; **SMD:** standardised mean difference

#### Explanations

a. Lack of blinding for participants.

b. Significant heterogeneity in the results.

c. Results were not robust: excluding specific studies altered the statistical significance.

d. Suspected publication bias

e. The 95% confidence intervals ranged from 1.26 to 11.53.

f. The 95% confidence intervals were wide and ranged from -2.40 to 11.29.
