# Supplementary material for: Effectiveness of Mobile Health Interventions in Pediatric Cancer: Systematic Review and Meta-Analysis of Randomized Controlled Trials
Source: JMIR Mhealth Uhealth. 2026 Apr 22;14:e86836. doi: 10.2196/86836 (PMC13102325; doi:10.2196/86836)
Supplement: Multimedia Appendix 3 [file mhealth-v14-e86836-s003.docx]

**Figure S1.** Sensitivity analysis: infection incidence

**Figure S2.** Sensitivity analysis: quality of life

**Figure S3.** Sensitivity analysis: overall PICC-related complications

**Figure S4.** Sensitivity analysis: PICC puncture site infection

**Figure S5.** Sensitivity analysis: PICC phlebitis

**Figure S6.** Sensitivity analysis: PICC thrombogenesis

**Figure S7.** Sensitivity analysis: PICC puncture site bleeding

**Figure S8.** Sensitivity analysis: PICC catheter occlusion

**Figure S9.** Sensitivity analysis: PICC catheter dislodgement

**Figure S10.** Sensitivity analysis: PICC catheter displacement

**Figure S11.** Sensitivity analysis: PICC treatment adherence
