# Supplementary material for: Effectiveness of Mobile Health Interventions in Pediatric Cancer: Systematic Review and Meta-Analysis of Randomized Controlled Trials
Source: JMIR Mhealth Uhealth. 2026 Apr 22;14:e86836. doi: 10.2196/86836 (PMC13102325; doi:10.2196/86836)
Supplement: Multimedia Appendix 4 [file mhealth-v14-e86836-s004.docx]

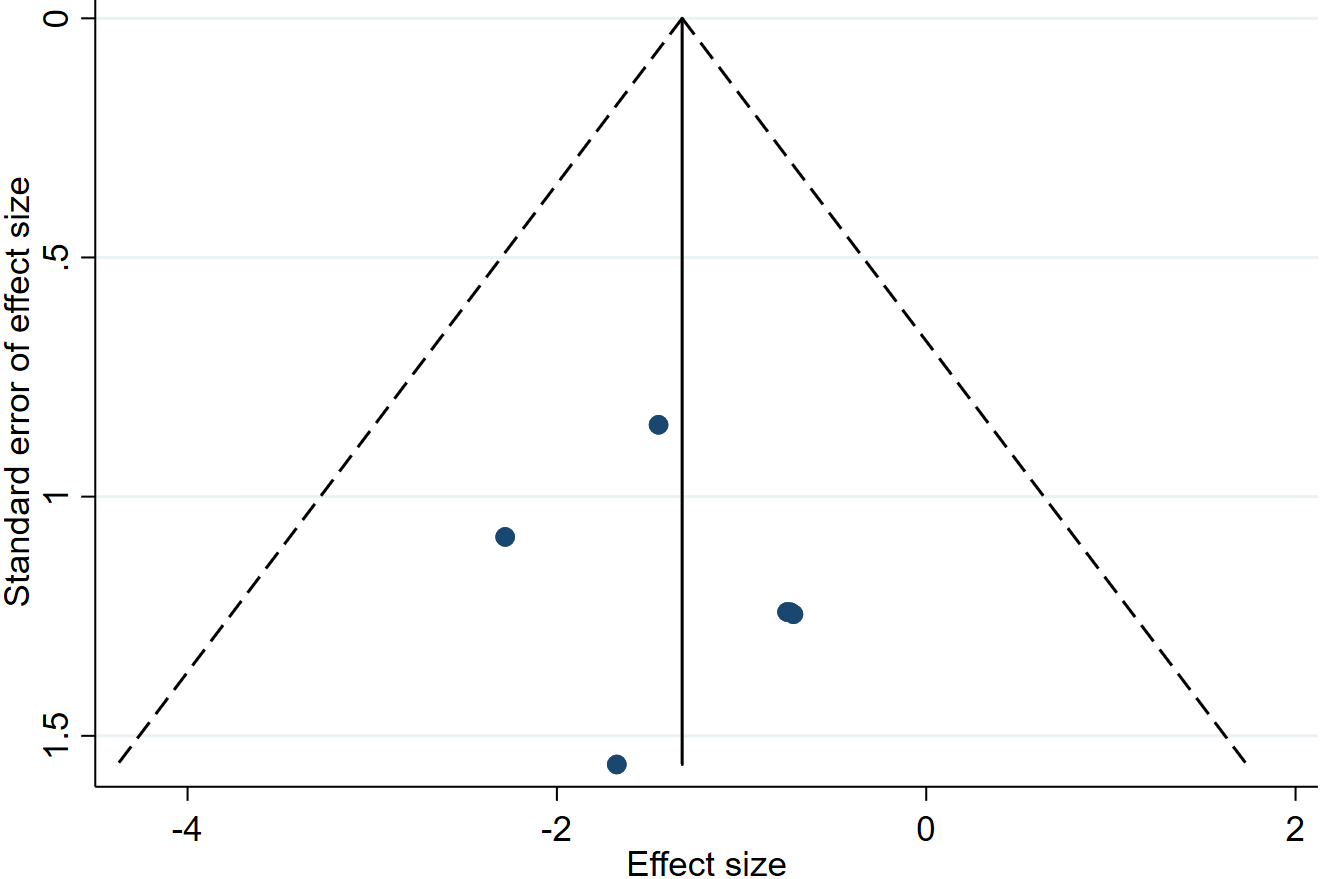


**Figure S1.** Funnel plots: infection incidence


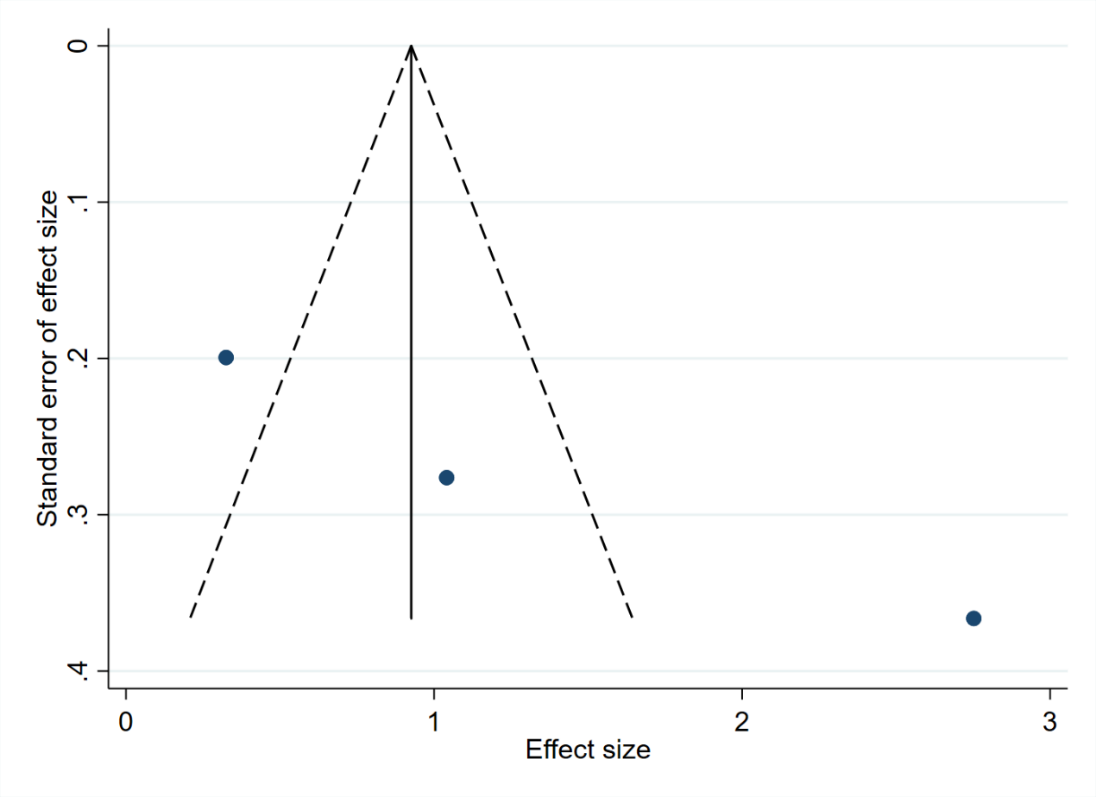


**Figure S2.** Funnel plots: quality of life


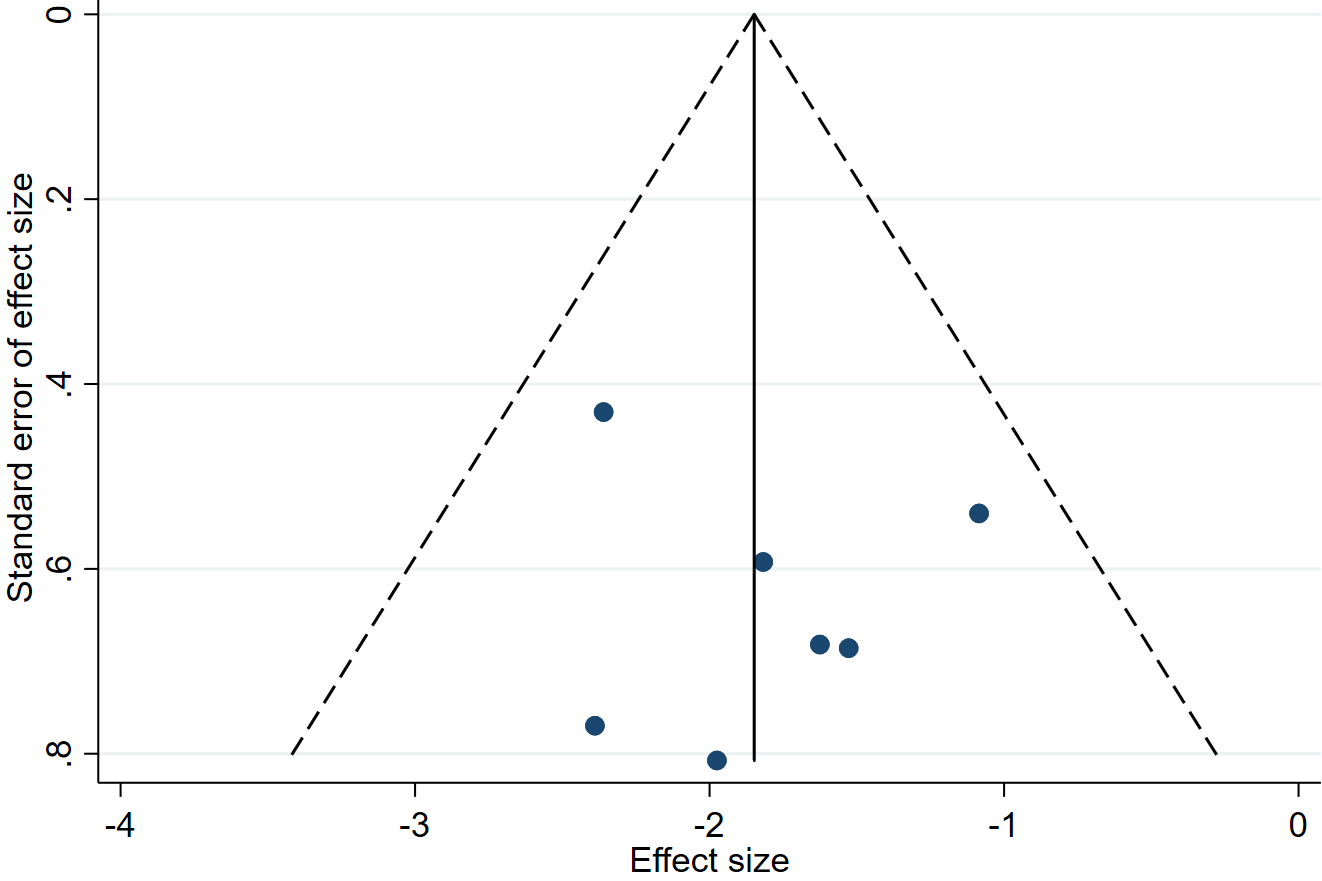


**Figure S3.** Funnel plots: overall PICC-related complications


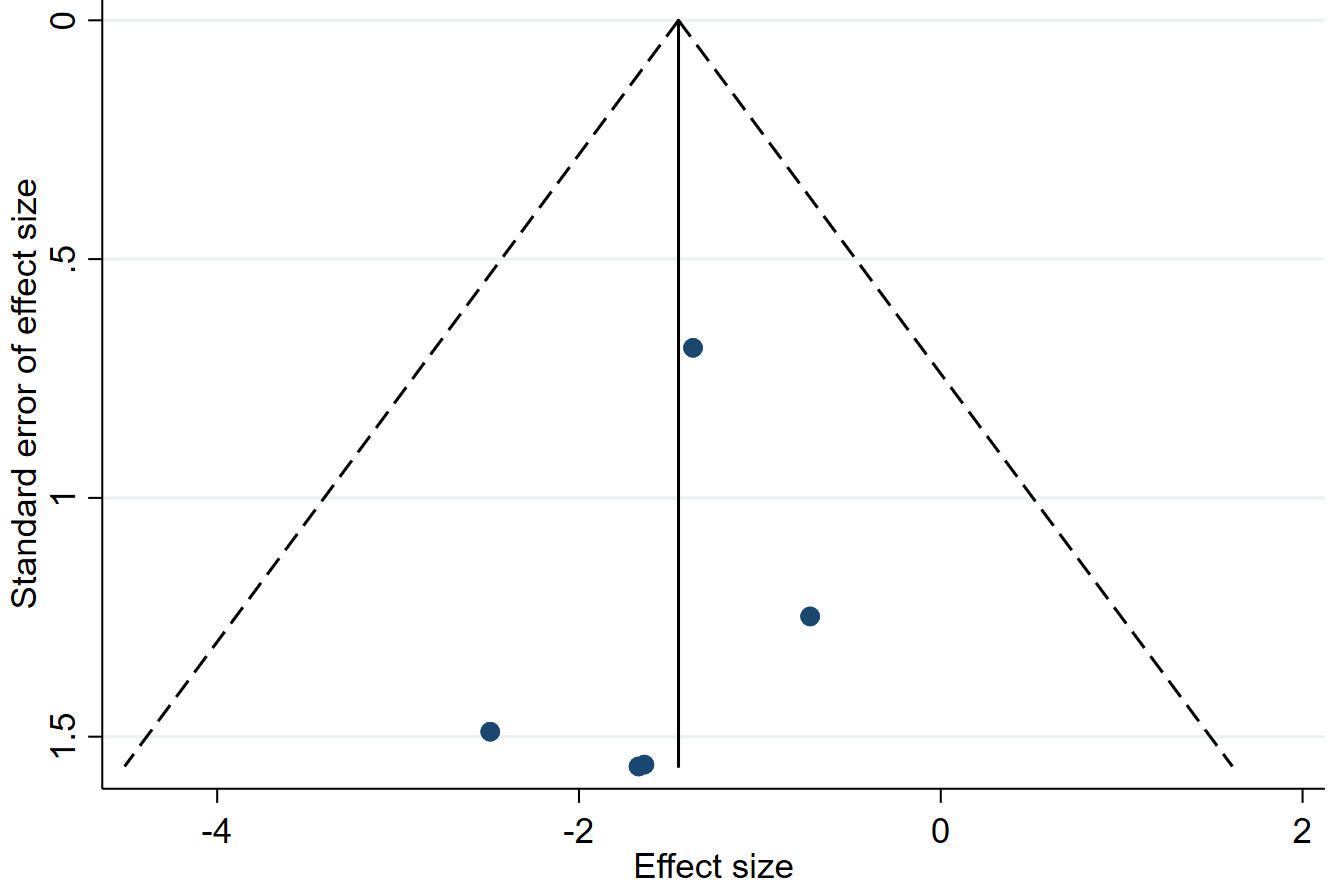


**Figure S4.** Funnel plots: PICC puncture site infection


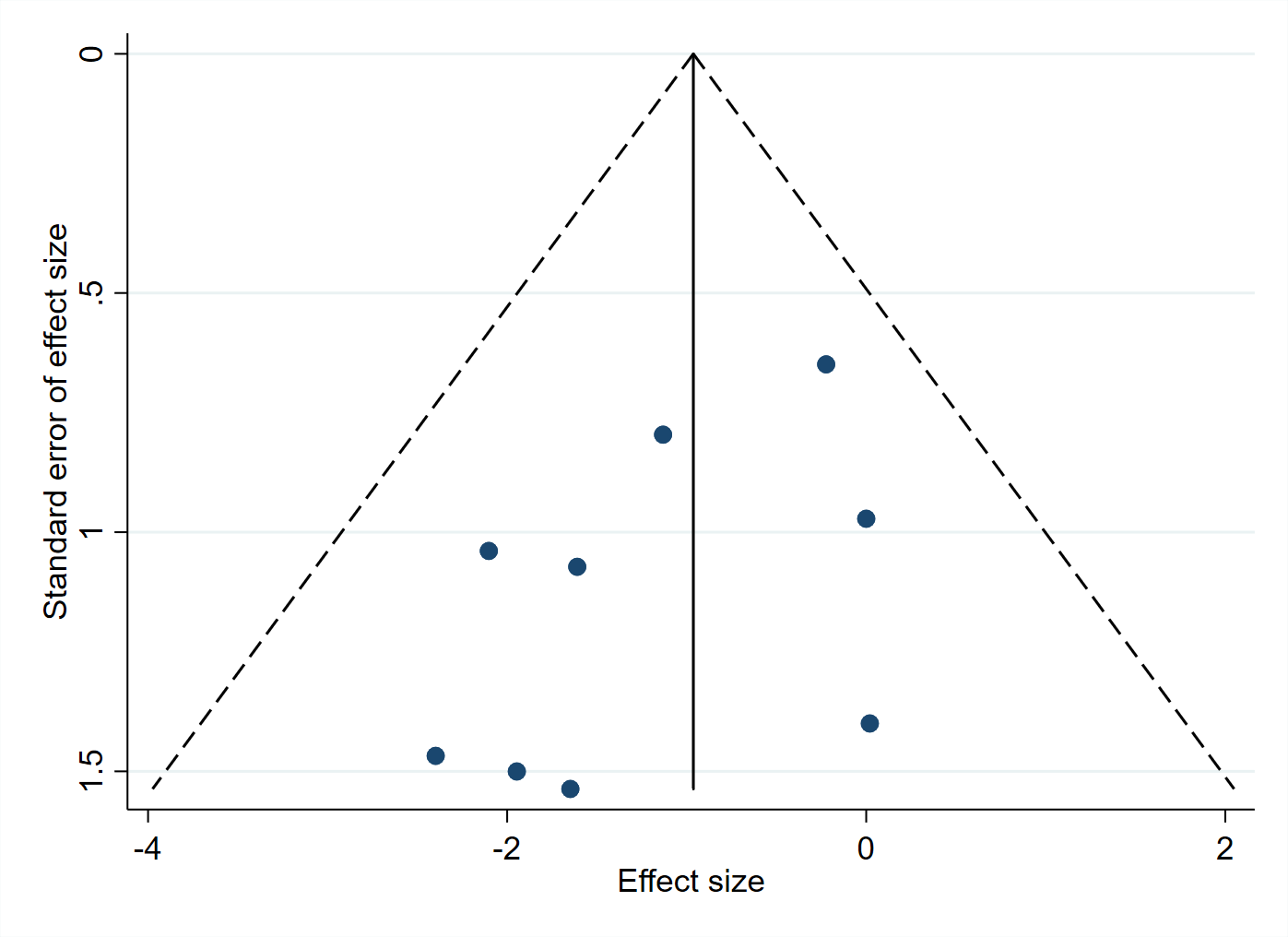


**Figure S5.** Funnel plots: PICC phlebitis


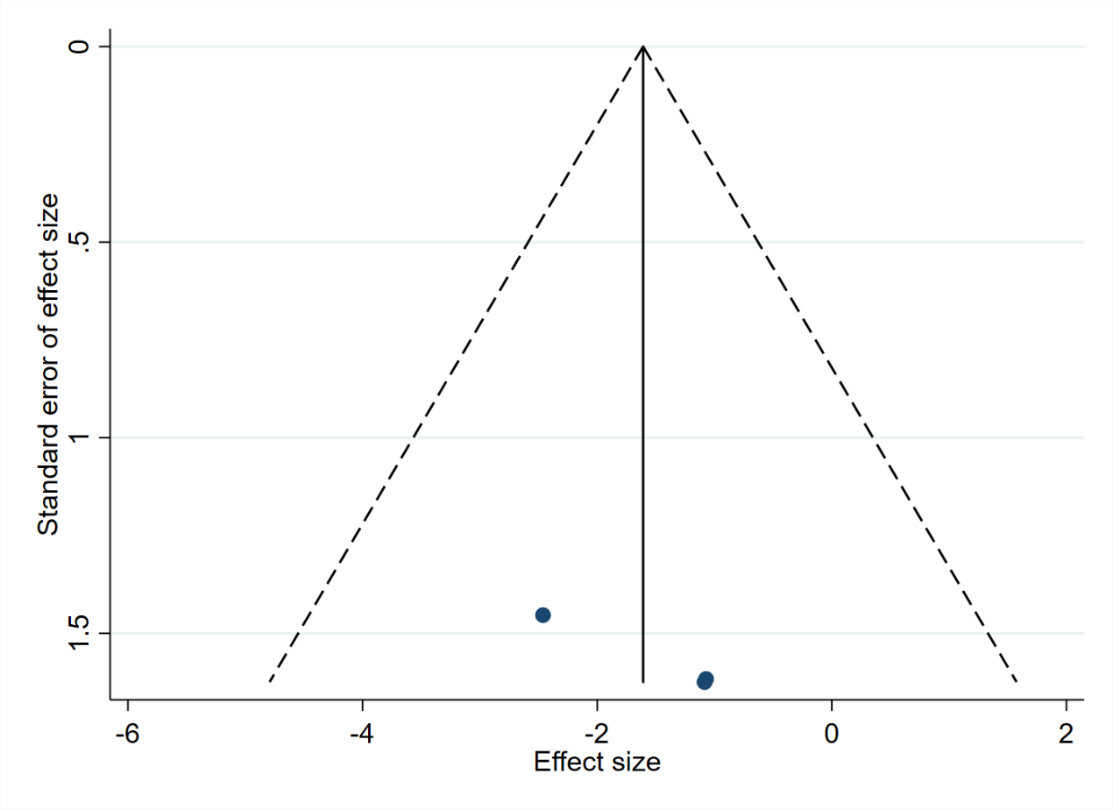


**Figure S6.** Funnel plots: PICC thrombogenesis


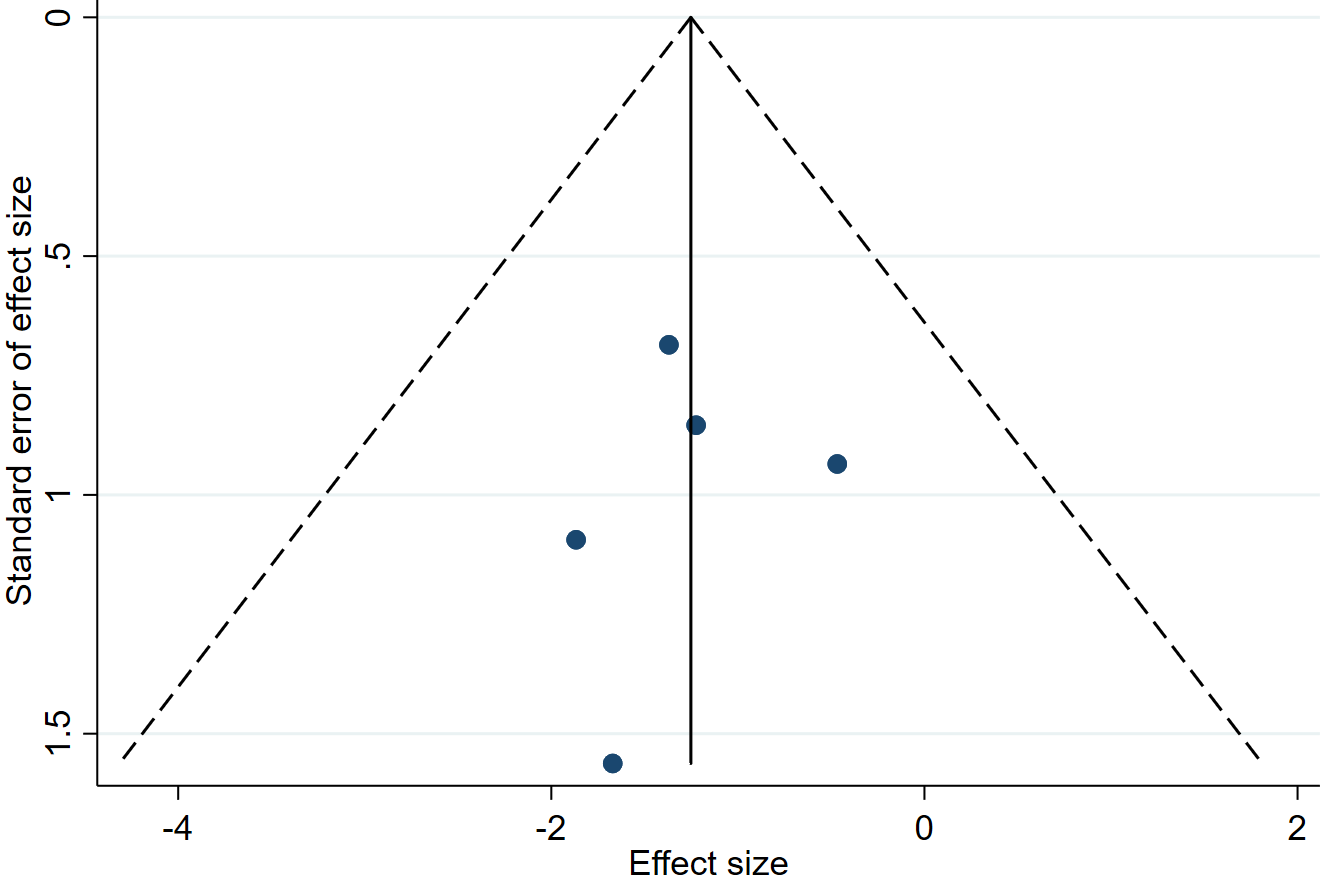


**Figure S7.** Funnel plots: PICC puncture site bleeding


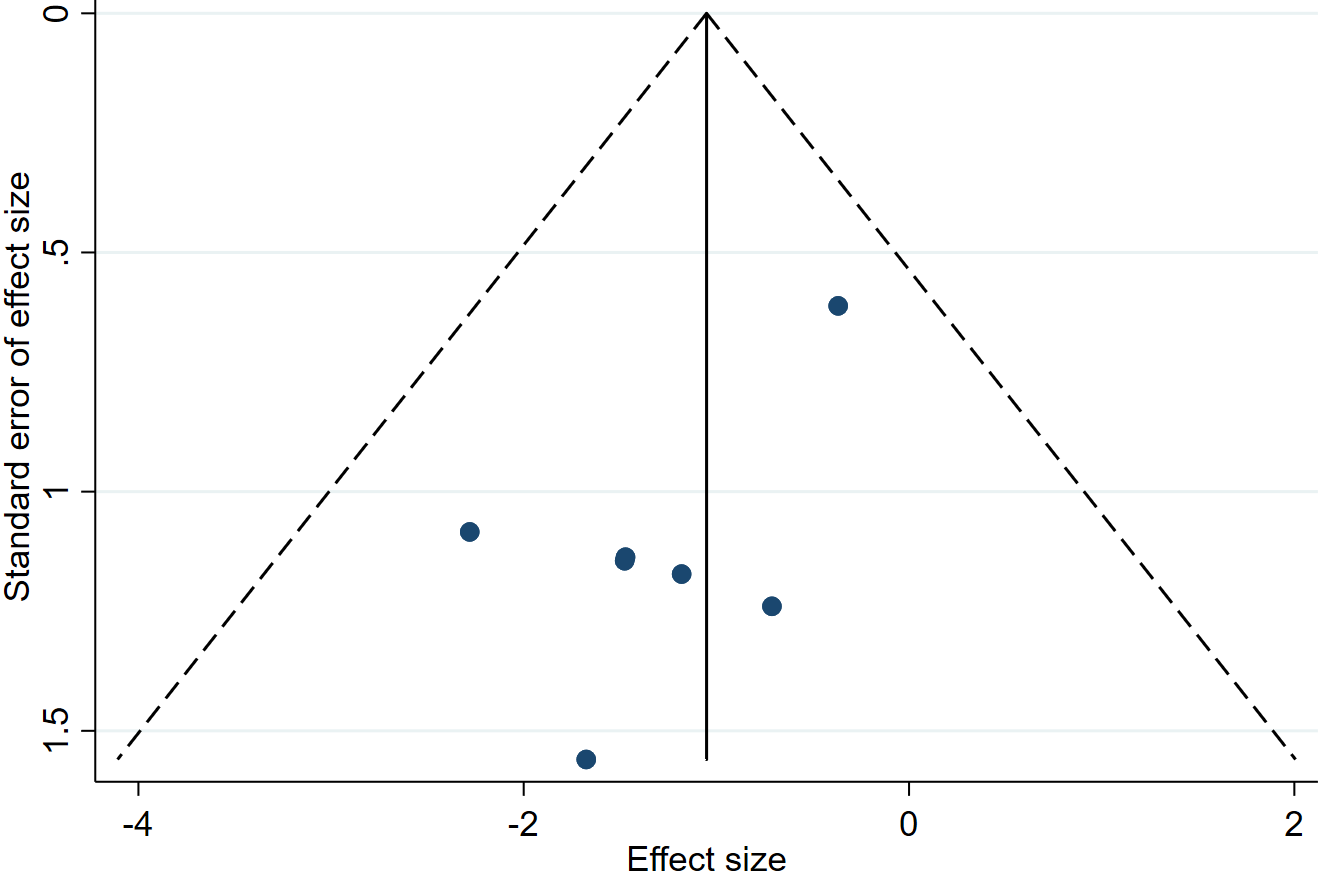


**Figure S8.** Funnel plots: PICC catheter occlusion


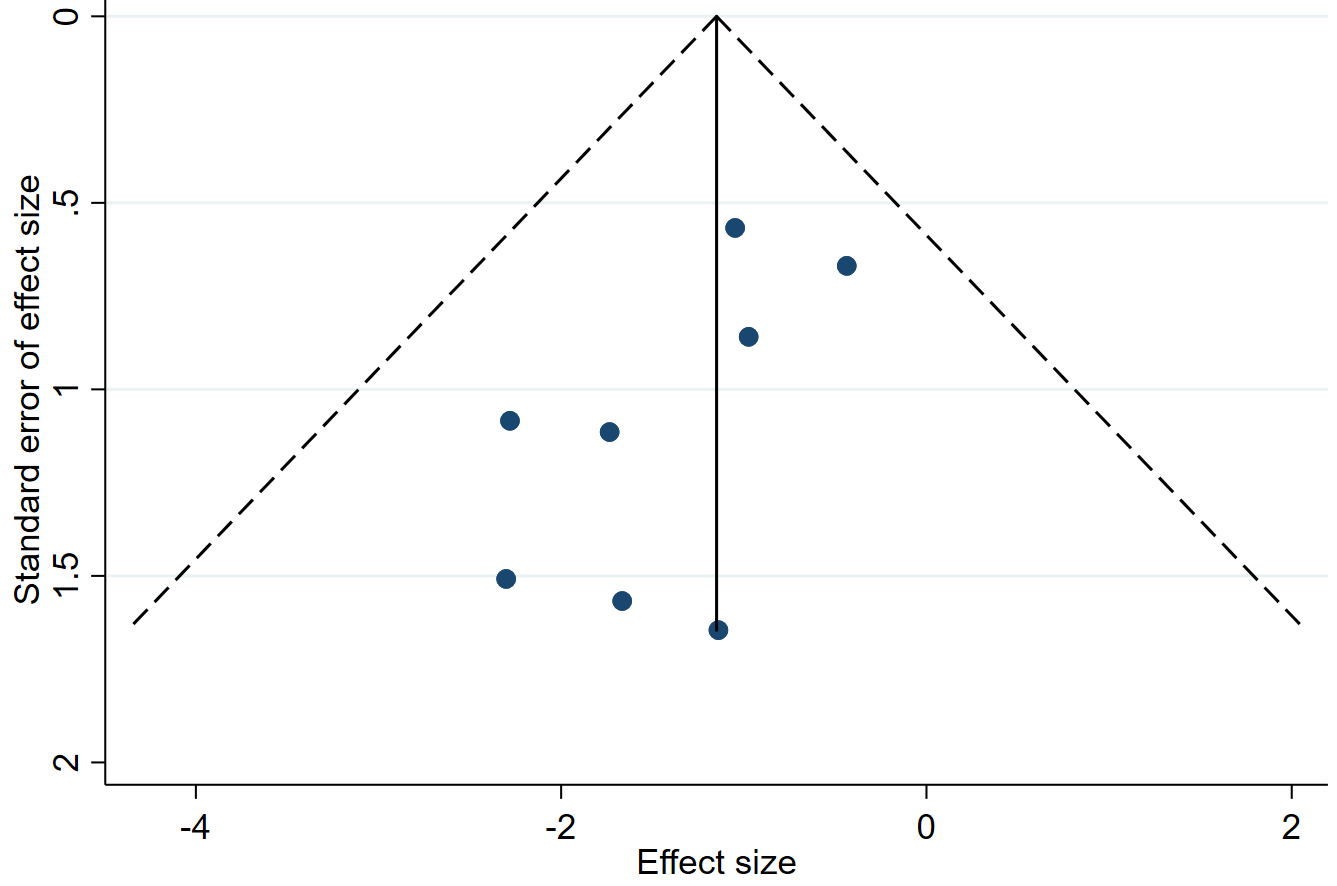


**Figure S9.** Funnel plots: PICC catheter dislodgement


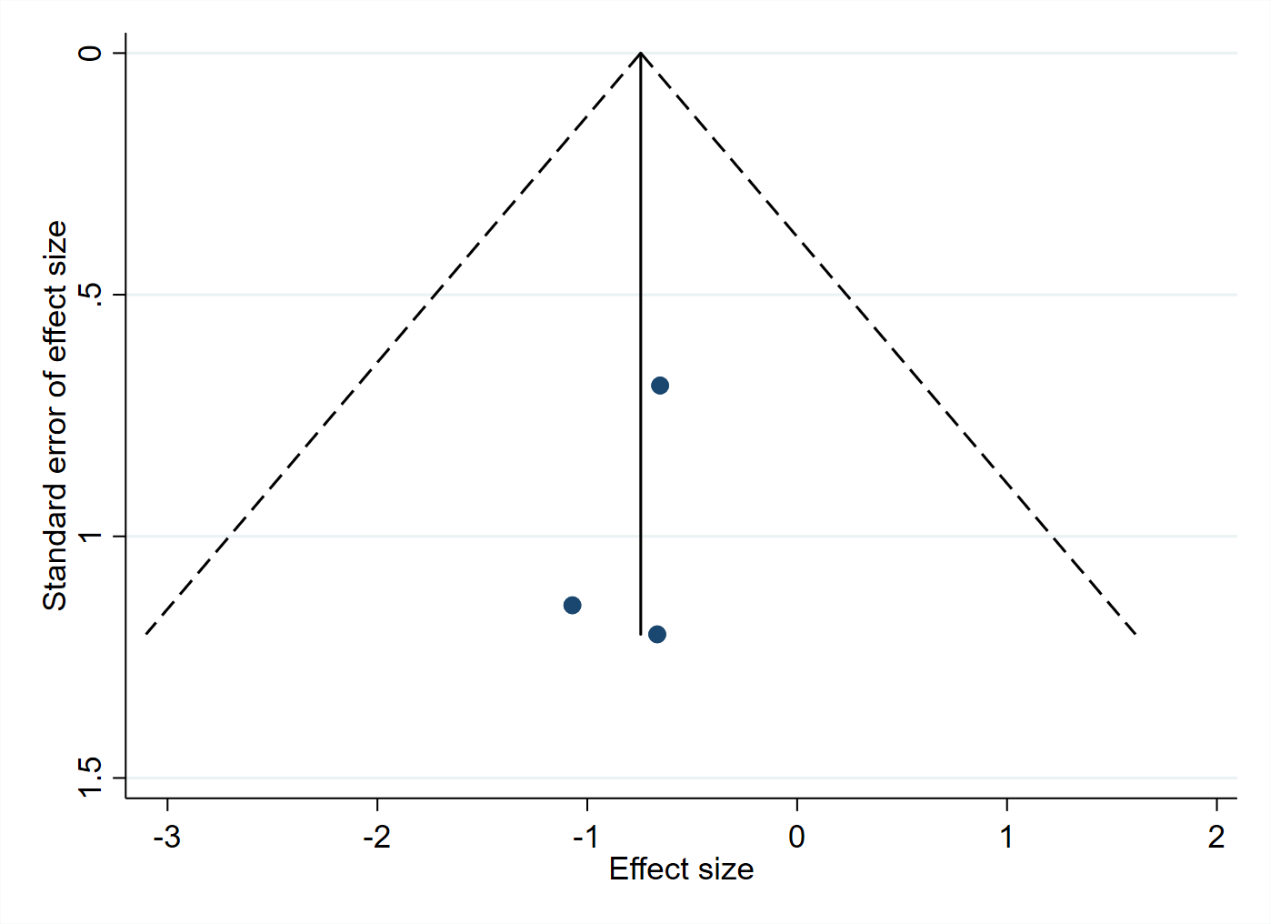


**Figure S10.** Funnel plots: PICC catheter displacement


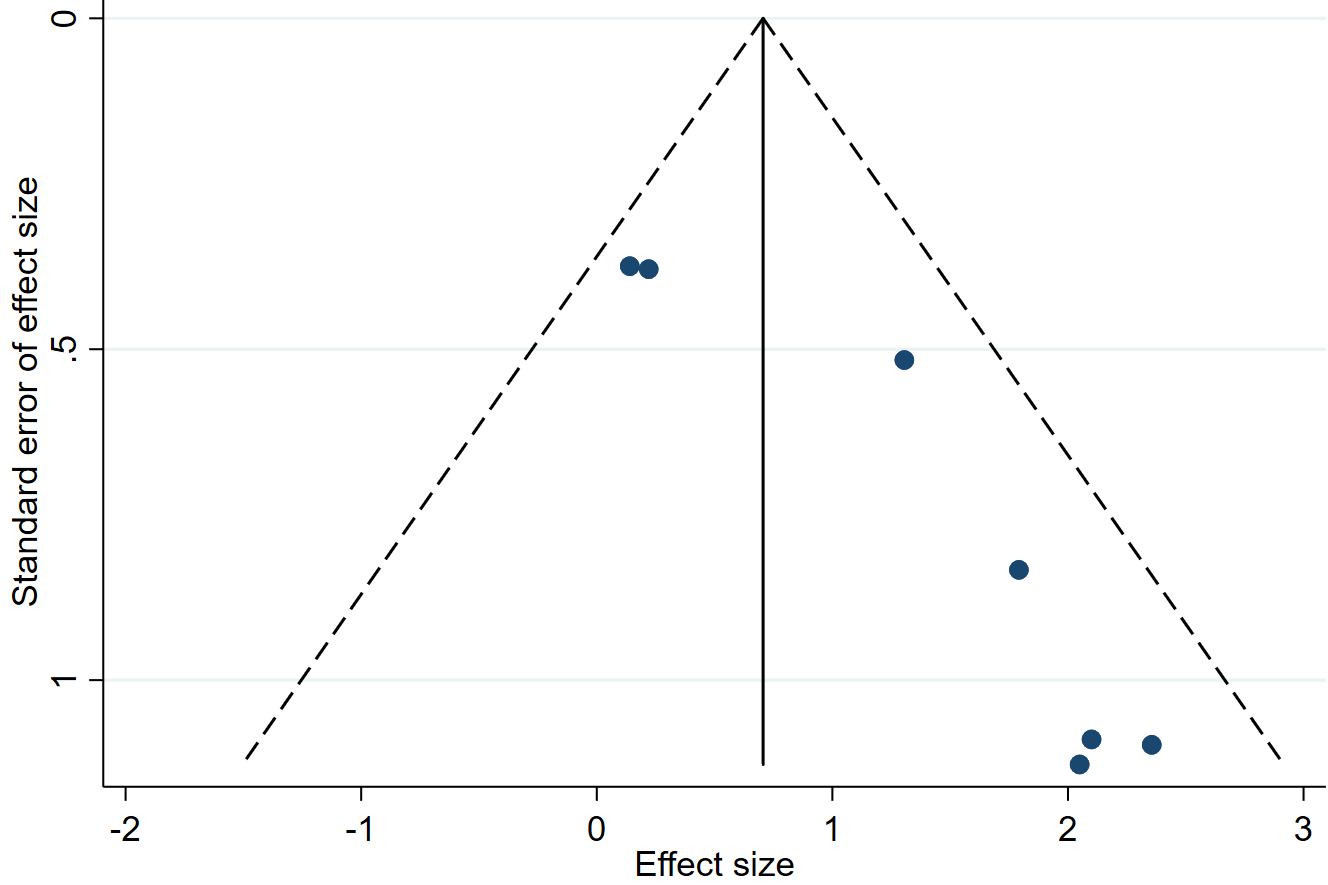


**Figure S11.** Funnel plots: PICC treatment adherence


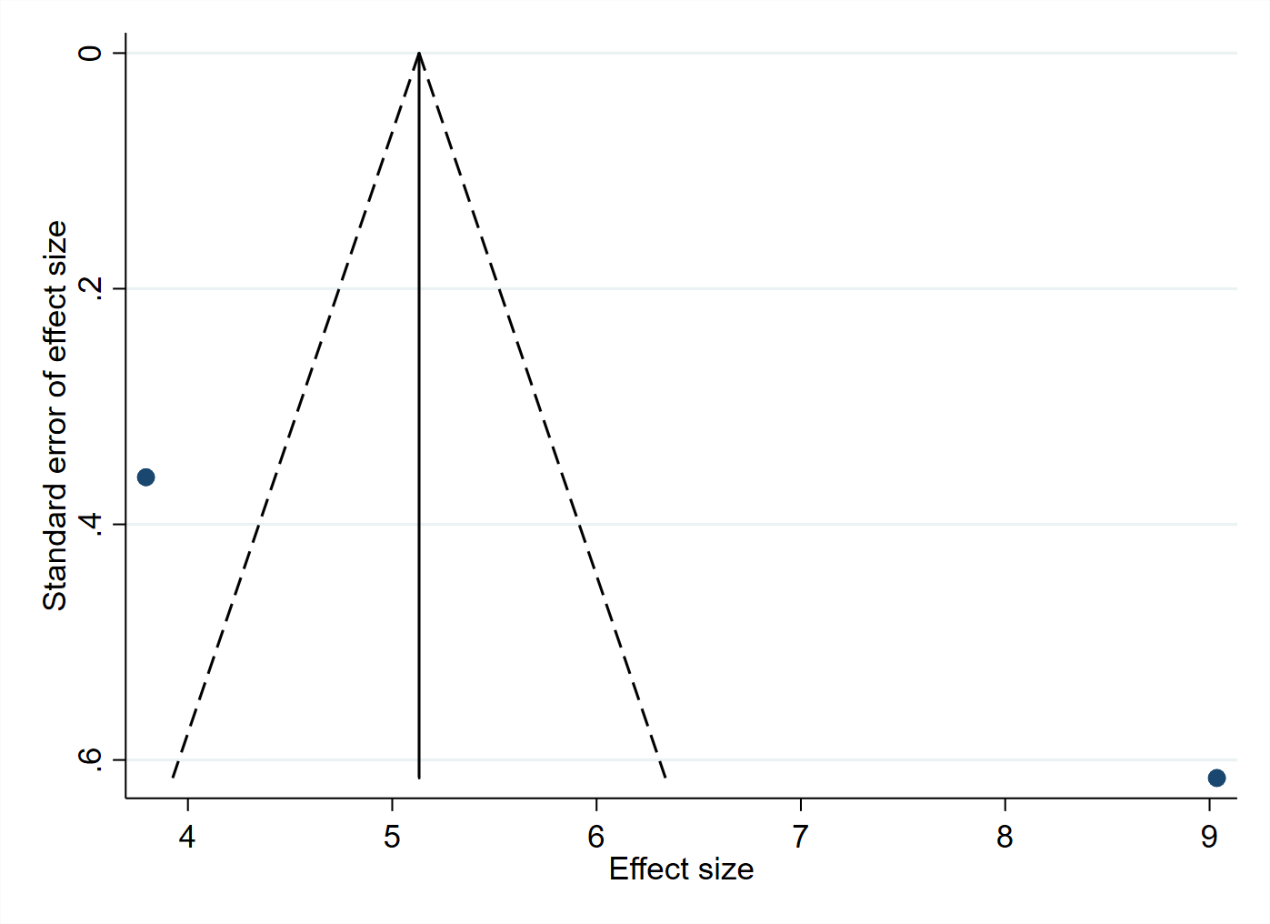


**Figure S12.** Funnel plots: self-management ability


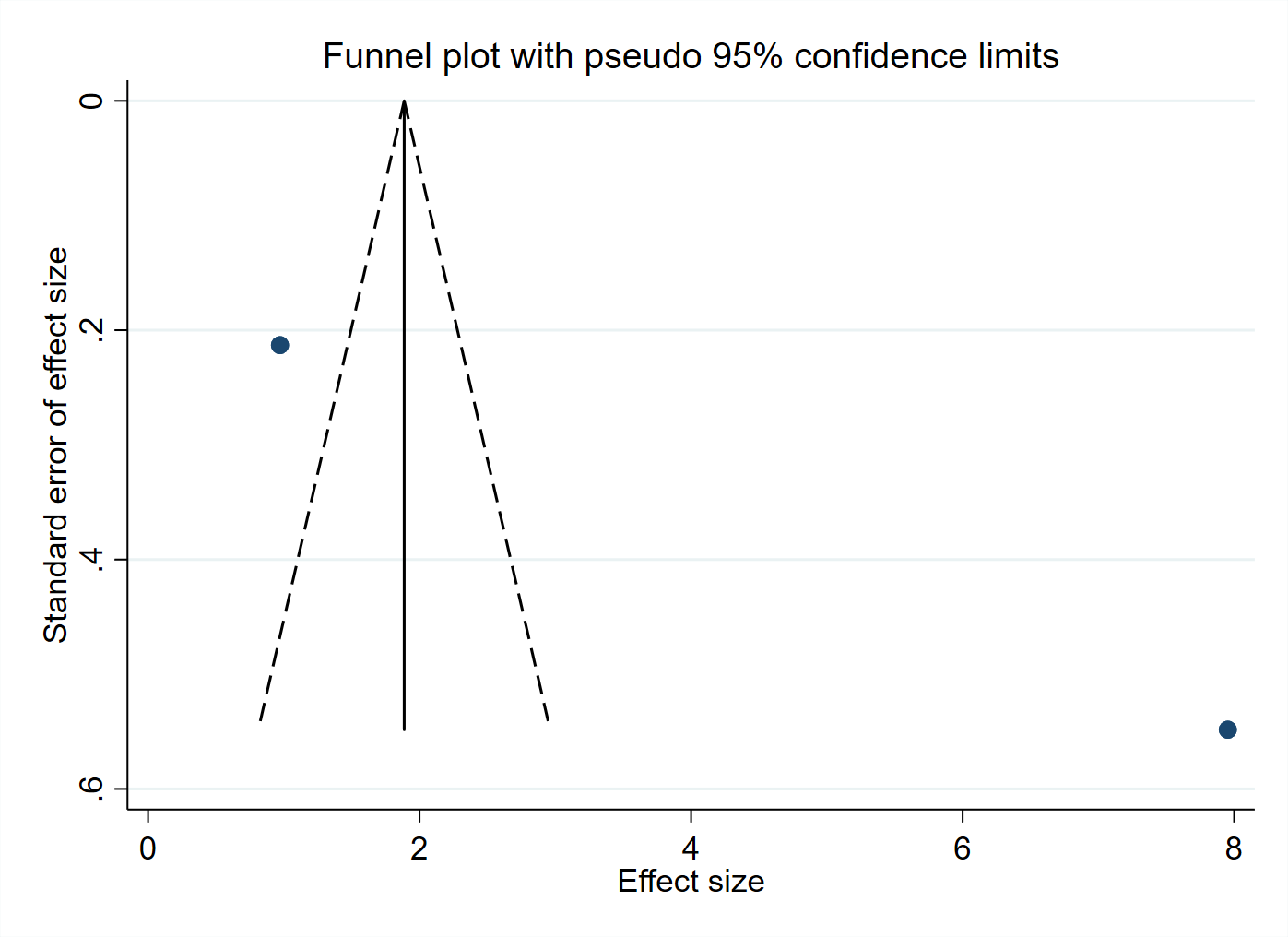


**Figure S13.** Funnel plots: health knowledge
